# Ambient Air Pollution and Hospitalizations for Schizophrenia in China

**DOI:** 10.1001/jamanetworkopen.2024.36915

**Published:** 2024-10-02

**Authors:** Lijun Bai, Yunxing Jiang, Kai Wang, Cuiyao Xie, Hairong Yan, Yu You, Huimeng Liu, Juan Chen, Jinxi Wang, Chen Wei, Yinxiang Li, Jian Lei, Hong Su, Shiquan Sun, Furong Deng, Xinbiao Guo, Shaowei Wu

**Affiliations:** 1 Department of Occupational and Environmental Health, School of Public Health, Xi’an Jiaotong University Health Science Center, Xi’an, Shaanxi, China; 2 Key Laboratory of Environment and Genes Related to Diseases, Ministry of Education, Xi’an, Shaanxi, China; 3 Key Laboratory for Disease Prevention and Control and Health Promotion of Shaanxi Province, Xi’an, Shaanxi, China; 4 Key Laboratory of Trace Elements and Endemic Diseases in Ministry of Health, Xi’an, Shaanxi, China; 5 Yunyi Health Technology Co Ltd, Beijing, China; 6 China-Europe Association for Technical and Economic Cooperation, Beijing, China; 7 Department of Epidemiology and Health Statistics, School of Public Health, Anhui Medical University, Hefei, Anhui, China; 8 School of Public Health, Xi’an Jiaotong University Health Science Center, Xi’an, Shaanxi, China; 9 Center for Single-Cell Omics and Health, Key Laboratory of Trace Elements and Endemic Diseases, Xi’an Jiaotong University, Xi’an, Shaanxi, China; 10 Department of Occupational and Environmental Health Sciences, School of Public Health, Peking University, Beijing, China

## Abstract

**Importance:**

Schizophrenia episodes may be triggered by short-term environmental stimuli. Short-term increases in ambient air pollution levels may elevate the risk of schizophrenia episodes, yet few epidemiologic studies have examined this association.

**Objective:**

To investigate whether short-term increases in air pollution levels are associated with an additional risk of schizophrenia episodes, independent of absolute air pollution concentrations, and whether sustained increases in air pollution levels for several days are associated with more pronounced risks of schizophrenia episodes.

**Design, Setting, and Participants:**

This nationwide, population-based, time-stratified case-crossover study was performed based on hospitalization records for schizophrenia across 295 administrative divisions of prefecture-level or above cities in China. Records were extracted from 2 major health insurance systems from January 1, 2013, to December 31, 2017. Thirty-six cities with a small number of schizophrenia hospitalizations (n < 50) were excluded. Data analysis for this study was performed from January to March 2024.

**Exposure:**

Daily absolute concentrations of fine particulate matter (PM_2.5_), inhalable particulate matter (PM_10_), nitrogen dioxide, sulfur dioxide, ozone, and carbon monoxide were collected. Air pollution increases between neighboring days (APINs) were generated as the differences in absolute air pollution concentrations on the current day minus that on the previous day. Sustained increases (APIN ≥5 μg/m^3^ for PM_2.5_ and PM_10_, APIN ≥1 μg/m^3^ for nitrogen dioxide and sulfur dioxide, and APIN ≥0.05 mg/m^3^ for carbon monoxide) lasting for 1 or more to 4 or more days were defined for different air pollutants.

**Main Outcome and Measure:**

Patients with schizophrenia episodes were identified by principal discharge diagnoses of schizophrenia. A conditional logistic regression model was used to capture the associations of absolute concentrations, APINs, and sustained increase events for different air pollutants with risks of schizophrenia hospitalizations.

**Results:**

The study included 817 296 hospitalization records for schizophrenia across 259 Chinese cities (30.6% aged 0-39 years, 56.4% aged 40-64 years, and 13.0% aged ≥65 years; 55.04% male). After adjusting for the absolute concentrations of respective air pollutants, per-IQR increases in 6-day moving average (lag_0-5_) APINs of PM_2.5_, PM_10_, nitrogen dioxide, sulfur dioxide, and carbon monoxide were associated with increases of 2.37% (95% CI, 0.88%-3.88%), 2.95% (95% CI, 1.46%-4.47%), 4.61% (95% CI, 2.93%-6.32%), 2.16% (95% CI, 0.59%-3.76%), and 2.02% (95% CI, 0.39%-3.68%) in schizophrenia hospitalizations, respectively. Greater risks of schizophrenia hospitalizations were associated with sustained increases in air pollutants lasting for longer durations up to 4 or more days.

**Conclusions and Relevance:**

This case-crossover study of the association between ambient air pollution increases and schizophrenia hospitalizations provides novel evidence that short-term increases in ambient air pollution levels were positively associated with an elevated risk of schizophrenia episodes. Future schizophrenia prevention practices should pay additional attention to APINs, especially sustained increases in air pollution levels for longer durations, besides the absolute air pollution concentrations.

## Key Points

**Question:**

Are short-term increases in ambient air pollution levels associated with elevated risk of schizophrenia episodes?

**Findings:**

In this case-crossover study of 817 296 patients in China, short-term increases in ambient air pollution levels between neighboring days were positively associated with increased risks of schizophrenia hospitalizations independent of the absolute air pollution concentrations. Sustained increases in air pollution levels for a longer duration were associated with higher risks.

**Meaning:**

These findings suggest that relative increases in air pollution levels between neighboring days, especially sustained increases lasting for several days, may have the potential to trigger additional risks of schizophrenia episodes.

## Teaser

This case-crossover study of patients in China examines the association between short-term increases in air pollution levels and additional risk of hospitalizations for schizophrenia episodes.

## Introduction

Schizophrenia affects approximately 24 million people or 1 in 300 people (0.32%) worldwide, and this rate corresponds to 1 in 222 people (0.45%) among adults.^1^ Most patients with schizophrenia experience multiple relapses, which generally result in poorer longitudinal outcomes, including a greater risk of suicide and reduced quality of life.^2^ Ambient air pollution, the second highest risk factor for noncommunicable diseases, is likely to serve as one of the key risk factors for mental disorders.^3^

Acute episodes of schizophrenia are often linked to acute oxidative stress,^4^ which can be stimulated by short-term exposure to ambient air pollution.^5^ Existing studies investigating this association are limited to individual cities or specific subpopulations (such as patients aged ≥65 years).^6,7^ A comprehensive study covering all age groups that could systematically elucidate the association between short-term exposure to ambient air pollution and risk of schizophrenia episodes is still lacking.

Particularly, existing studies are primarily concentrated on the absolute concentrations of ambient air pollution,^6,7^ which are probably unable to capture the potential effects of short-term increases in air pollution concentrations before the onset of schizophrenia episodes. In fact, adverse mental health events (eg, acute schizophrenia episodes) may be more sensitive to air pollution increase within a short time, analogous to relocation stress syndrome, which refers to a state in which an individual experiences physiologic and/or psychosocial disturbances as a result of change in environment.^8^ However, no study has ever investigated the association of short-term increases in air pollution levels, such as relative increases in air pollution levels on the current day compared with the previous day, on risk of schizophrenia episodes. In addition, exposure to continuous increases in air pollution levels may generate accumulative oxidative damages,^9^ which supports the hypothesis that sustained increases in air pollution levels for several days are likely to be associated with a greater risk of acute schizophrenia episodes. Identifying associations of short-term relative increases in air pollution levels with schizophrenia episodes may help advance the understanding of air pollution–related risk of schizophrenia episodes and provide scientific basis for the formulation of targeted intervention strategies. Although air quality has improved considerably in China since 2013, current air pollution levels still have great health impacts.^10^ Additionally, because of the changes in the surface cyclone frequency brought on by climate change, air pollution events may become more frequent and severe and last longer.^11^ Investigation for the potential adverse health effects of short-term increases in air pollution levels is necessary.

We hypothesized that the air pollution increases between neighboring days (APINs) may be associated with an additional risk of schizophrenia episodes, independent of the absolute air pollution concentrations, and sustained increases in air pollution levels for 2 or more days may be associated with higher risks of schizophrenia episodes. Individuals who are undergoing very severe schizophrenia episodes generally require inpatient care at a hospital; thus, hospitalizations with primary diagnoses of schizophrenia are considered as acute schizophrenia episodes.^12,13,14^ This study aims to investigate the association among absolute concentrations, APINs, and sustained increases of different air pollutants and hospitalizations for schizophrenia in China.

## Methods

### Study Design

We conducted a time-stratified case-crossover study, which has been widely applied to evaluate the associations between short-term exposure to air pollution and health outcomes.^15,16^ For each city, we created a case-crossover dataset, in which a case-day was defined as the day of hospitalization for schizophrenia. Matched control-days were identified as days with the same city (same person) of the same week, month, and year. Such self-matching can control for the confounding variables that do not change markedly within a month or a year, including age, race, sex, body mass index, diet, educational level, income, and living situation or marital status. Matching by day of the week controlled for potential confounding that varies within a week, such as weekday and weekend differences in air pollution and hospitalizations, with bidirectional selection of control-days before and after the case-day within a month to remove potential bias induced by long-term time trends of air pollutants.^16^ In the time-stratified case-crossover design, participants served as their own control, and the estimation was generated by comparing the concentration of each air pollutant on or before the case-day with that on or before the control-days.^17^ This study was exempted from institutional review board approval by the Biomedical Ethics Committee of the Health Science Center of Xi’an Jiaotong University because all data used for this study were collected for administrative purposes without any individual identifiers. This report followed the Strengthening the Reporting of Observational Studies in Epidemiology (STROBE) reporting guideline.

### Air Pollution Exposures

In this time-stratified case-crossover study, daily absolute concentrations of 6 standard ambient air pollutants, including fine particulate matter (PM_2.5_), inhalable particulate matter (PM_10_), nitrogen dioxide, sulfur dioxide, ozone, and carbon monoxide, were collected from the National Urban Air Quality Real-time Publishing Platform^18^ issued by the China National Environmental Monitoring Centre (CNEMC), serving as indicators of population exposure to ambient air pollution.^19,20^ City-specific daily 24-hour concentrations of PM_2.5_, PM_10_, sulfur dioxide, nitrogen dioxide, and carbon monoxide and daily maximum 8-hour mean concentrations of ozone were averaged across all monitoring stations in each city if there was more than 1 monitor in that city. Missing air pollution data (missing rate, 11.17%) were filled in by the high-resolution Chinese air quality reanalysis dataset, which was produced by assimilating surface observations of air pollution concentrations retrieved from the CNEMC.^21^ The intraclass correlation coefficients between daily concentrations of different air pollutants from the CNEMC and the Chinese air quality reanalysis dataset ranged from 0.66 to 0.84 in this study (eTable 1 in Supplement 1).

### Definitions of APINs and Sustained Increase Events for Different Air Pollutants

APIN was defined as the difference in daily air pollution concentrations on the current day minus that on the previous day. Therefore, APIN is a continuous variable, including both positive and negative values. Different increase units were set for different air pollutants based on the numerical magnitude in concentration distributions of different air pollutants. APINs of 5 μg/m^3^ or greater lasting for 1 day or more, 2 days or more, 3 days or more, and 4 days or more were defined as sustained increase events for PM_2.5_ and PM_10_; APINs of 1 μg/m^3^ or more lasting for 1 day or more, 2 days or more, 3 days or more, and 4 days or more were defined as sustained increase events for sulfur dioxide and nitrogen dioxide; and APINs of 0.05 mg/m^3^ or more lasting for 1 day or more, 2 days or more, 3 days or more, and 4 days or more were defined as sustained increase events for carbon monoxide. Sustained increase events longer than 5 days were not considered because of few such events.

### Health Data

Hospitalization records for schizophrenia were extracted from the urban employee-based basic medical insurance scheme (UEBMI) and the urban resident-based basic medical insurance scheme (URBMI), 2 major health insurance systems in the Chinese mainland.^22^ By 2014, China’s universal medical insurance systems had covered 97.5% of the entire population.^23^

Daily hospitalization data across 295 administrative divisions of prefecture-level or above cities (of 338 such cities in total in 2017^24^) in the Chinese mainland were accessibly extracted from January 1, 2013, to December 31, 2017. To ensure a reliable analysis, 36 cities with a small number of schizophrenia hospitalizations (n < 50) were excluded. Finally, we included data from a total of 259 cities, among which 244 cities had data available from the UEBMI, 227 cities had data available from the URBMI, and 212 cities had data from both the UEBMI and URBMI.

### Outcome Definition

All daily hospitalizations with principal discharge diagnoses coded as F20 (a valid *International Statistical Classification of Diseases, Tenth Revision, Clinical Modification* diagnosis code meaning schizophrenia) were included in this study. In China, the principal discharge diagnosis is identified as the disease that is mainly treated during hospitalization and is generally regarded as the primary cause of hospitalization.

### Covariates

In the main models, time-variant meteorologic data (daily ambient air surface temperature and relative humidity) in each city were obtained from the China Meteorological Data Sharing Service System.^25^ Information on public holidays was collected from holiday arrangement issued by the General Office of the State Council during 2013 to 2017 to control for the potential influence of holidays. The other variables used in the subgroup and meta-regression analyses are described in eMethods 1 in Supplement 1.

### Statistical Analysis

A 2-stage analytical approach was applied to evaluate the city-specific and overall associations between ambient air pollution and daily hospitalizations for schizophrenia.^26^ In the first stage, a conditional logistic regression model was used to estimate city-specific percentage changes in hospitalizations for schizophrenia associated with per-IQR increases in the absolute concentrations and APINs of each air pollutant. Detailed model settings are described in eMethods 2 in Supplement 1. To further capture the excess hospitalization risk and burden associated with exposure to air pollution levels above the World Health Organization (WHO) air quality guideline (AQG), excessive and heavily excessive air pollution levels were defined according to the 24-hour mean or daily maximum 8-hour mean concentration limits of the WHO-AQG or specific interim target levels. Details about the definitions are provided in eMethods 3 in Supplement 1.

In the second stage, the overall associations across all the included cities were obtained by pooling the city-specific associations using the random-effects model. The overall exposure-response curves at the representative time windows were plotted for the associations following the approach developed by Gasparrini et al.^27^ The associated overall attributable numbers and attributable fractions for hospitalizations and length of hospital stay based on the pooled associations were estimated during the study period (eMethods 4 in Supplement 1). The excess admissions and lengths of stay due to air pollution concentrations exceeding different WHO-AQGs were also evaluated (eMethods 5 in Supplement 1). Details about exploratory analyses for the modification by urbanization and mental health service level, subgroup analyses, meta-regression analyses, and sensitivity analyses are provided in eMethods 6 to 8 in Supplement 1.

All statistical analyses were conducted in R, version 4.0.5 (R Foundation), and the effect estimates were expressed as percentage changes with 95% CIs converted from relative risks in daily schizophrenia hospitalizations associated with per-IQR increases in absolute air pollution concentrations and APINs, respectively. We defined a dichotomous variable with 1 coding for the sustained increase events and 0 coding for the days not matching the definition for each air pollutant to evaluate the association of sustained increase events for a given air pollutant with hospitalizations for schizophrenia. To address the issue of multiple comparison, we used the Benjamini-Hochberg procedure to adjust the *P* values obtained for absolute concentrations and APINs of different air pollutants from the regression models for each specific time window. A 2-sided *P* < .05 was considered statistically significant. Data analysis for this study was performed from January to March 2024.

## Results

### Study Population Characteristics and Ambient Air Pollution Distribution

A total of 817 296 hospitalization records for schizophrenia (30.6% aged 0-39 years, 56.4% aged 40-64 years, and 13.0% aged ≥65 years; 55.04% male and 44.96% female) were extracted from the UEBMI and URBMI databases across 259 cities of prefecture-level or above in China (eTable 2 in Supplement 1). Patients enrolled in the UEBMI (56.07%) accounted for a higher proportion than those enrolled in the URBMI (43.93%). The central locations of the included 259 cities are shown in eFigure 1 in Supplement 1, and the detailed city list is given in eTable 3 in Supplement 1. Ambient levels of different air pollutants, meteorologic factors, and APINs during 2013 to 2017 are given in Table 1. The median (IQR) daily concentrations of air pollutants were 38.84 (23.00-62.33) μg/m^3^ for PM_2.5_, 64.93 (40.26-102.94) μg/m^3^ for PM_10_, 23.62 (13.64-37.00) μg/m^3^ for nitrogen oxide, 16.01 (9.33-27.67) μg/m^3^ for sulfur dioxide, 74.25 (51.90-100.38) μg/m^3^ for ozone, and 0.83 (0.57-1.18) mg/m^3^ for carbon monoxide. The Spearman correlation coefficients between air pollutants and meteorologic factors ranged from −0.24 to 0.90 (eTable 4 in Supplement 1), and the correlation coefficients between absolute air pollution concentrations and corresponding APINs ranged from 0.24 to 0.33 (eTable 5 in Supplement 1).

**Table 1.** Summary Statistics of Daily Ambient Air Pollutants and Meteorologic Variables in 259 Chinese Cities, 2013-2017

Variable	Mean (SD)	Median (IQR)
PM_2.5_, μg/m^3^	49.40 (41.72)	38.84 (23.00 to 62.33)
PM_10_, μg/m^3^	81.15 (69.78)	64.93 (40.26 to 102.94)
Nitrogen dioxide, μg/m^3^	27.29 (18.75)	23.62 (13.64 to 37.00)
Sulfur dioxide, μg/m^3^	23.28 (25.35)	16.01 (9.33 to 27.67)
8-Hour ozone, μg/m^3^	78.65 (37.31)	74.25 (51.90 to 100.38)
Carbon monoxide, mg/m^3^	0.97 (0.65)	0.83 (0.57 to 1.18)
APIN, μg/m^3^		
PM_2.5_	0.00 (28.32)	0.72 (−8.40 to 10.13)
PM_10_	0.00 (50.33)	1.06 (−12.98 to 15.45)
Nitrogen dioxide	0.00 (9.95)	0.07 (−3.76 to 4.22)
Sulfur dioxide	0.00 (14.40)	0.00 (−3.24 to 3.43)
8-Hour ozone	0.00 (24.29)	0.43 (−10.91 to 11.82)
Carbon monoxide, mg/m^3^	0.00 (0.37)	0.00 (−0.10 to 0.12)
Temperature, °C	13.91 (11.43)	16.00 (6.80 to 22.80)
Relative humidity, %	68.31 (18.15)	71.00 (57.00 to 82.00)

Abbreviations: APIN, air pollution increases between neighboring days; PM_10_, particulate matter with an aerodynamic diameter of 10 μm or less; PM_2.5_, particulate matter with an aerodynamic diameter of 2.5 μm or less.

### Associations of Absolute Air Pollution Concentrations With Hospitalizations for Schizophrenia and Potential Modification by Urbanization

**Figure 1.**
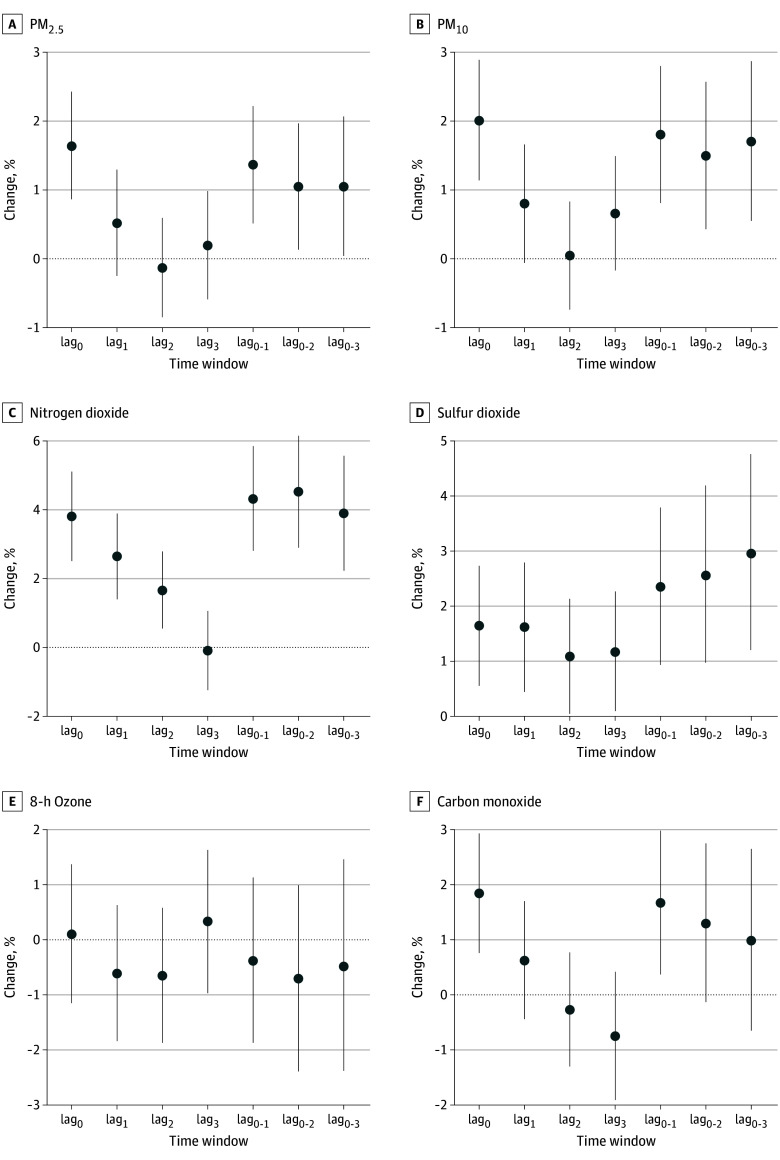
Overall Percentage Changes With 95% CIs in Daily Hospitalizations for Schizophrenia per-IQR Increase in Ambient Air Pollution Concentrations at Different Time Windows in 259 Chinese Cities, 2013-2017 Lag_0_ indicates current day; lag_1_, 1 day before; lag_2_, 2 days before; lag_3_, 3 days before; lag_0-1_, 2-day moving average of lag_0_ and lag_1_ days; lag_0-2_, 3-day moving average of lag_0_, lag _1_, and lag_2_ days; lag_0-3_, 4-day moving average of lag_0_, lag_1_, lag_2_, and lag_3_ days. Error bars indicate 95% CIs. PM_10_ indicates particulate matter with an aerodynamic diameter of 10 μm or less; PM_2.5_, particulate matter with an aerodynamic diameter of 2.5 μm or less.

Figure 1 shows that per-IQR increases in 2-day moving average (lag_0-1_) PM_2.5_, PM_10_, nitrogen dioxide, sulfur dioxide, and carbon monoxide were significantly associated with increases of 1.36% (95% CI, 0.51%-2.21%; adjusted *P* = .002), 1.80% (95% CI, 0.81%-2.80%; adjusted *P* = .001), 4.32% (95% CI, 2.81%-5.85%; adjusted *P* < .001), 2.35% (95% CI, 0.93%-3.79%; adjusted *P* = .002), and 1.67% (95% CI, 0.37%-2.98%; adjusted *P* = .01) in schizophrenia hospitalizations, respectively. Accordingly, per-5-μg/m^3^ increases in 2-day moving average (lag_0-1_) PM_2.5 _and per-5-ppb increases in 2-day moving average (lag_0-1_) nitrogen dioxide were associated with increases of 0.17% (95% CI, 0.07%-0.28%; adjusted *P* = .002) and 1.87% (95% CI, 1.22%-2.53%; adjusted *P* < .001) in schizophrenia hospitalizations, respectively. The exposure-response curves appeared to be generally linear over the absolute concentration ranges of these air pollutants (eFigure 2 in Supplement 1). No apparent association of 8-hour ozone with hospitalizations for schizophrenia were observed, consistent with the exposure-response curve for 8-hour ozone. Therefore, we mainly focused on the other air pollutants in the following analyses. Table 2 indicates that air pollution levels exceeding the current AQGs were associated with significant increases in schizophrenia hospitalizations. Notably, exposure to carbon monoxide (0.6-1.4 mg/m^3^) below the current AQG for 24-hour carbon monoxide (4 mg/m^3^) was also associated with an increase in hospitalizations for schizophrenia.

**Table 2.** Overall Percentage Changes With 95% CIs in Daily Hospitalizations for Schizophrenia Associated With Excessive or Heavily Excessive Ambient Air Pollution Concentrations Under Different Air Quality Guidelines in 259 Chinese Cities, 2013-2017

Air pollutant	Reference concentration	Excessive or heavily excessive concentration	Change (95% CI), %^a^
PM_2.5_	<25 μg/m^3^^b^	25-74 μg/m^3^	0.88 (0.17-1.60)
		≥75 μg/m^3^^c^	1.20 (0.18-2.20)
PM_10_	<45 μg/m^3^^d^	45-99 μg/m^3^	1.31 (0.60-2.00)
		≥100 μg/m^3^^e^	1.54 (0.63-2.50)
Nitrogen dioxide	<25 μg/m^3^^d^	25-49 μg/m^3^	3.39 (2.55-4.23)
		≥50 μg/m^3^^e^	4.34 (3.24-5.44)
Sulfur dioxide	<20 μg/m^3^^f^	20-39 μg/m^3^	2.45 (1.67-3.23)
		≥40 μg/m^3^^d^	3.43 (1.99-4.90)
Carbon monoxide	<0.6 mg/m^3^^g^	0.6-1.4 mg/m^3^	1.80 (0.86-2.67)
		≥1.5 mg/m^3^^g^	2.30 (0.98-3.70)

Abbreviations: PM_10_, particulate matter with an aerodynamic diameter of 10 μm or less; PM_2.5_, particulate matter with an aerodynamic diameter of 2.5 μm or less.

^a^ The reference category for each air pollution was the days with low daily air pollution concentration (reference concentration).

^b^ World Health Organization air quality guideline 2021 interim target 4 (24-hour mean).

^c^ World Health Organization air quality guideline 2021 interim target 1 (24-hour mean).

^d^ World Health Organization air quality guideline 2021 (24-hour mean).

^e^ World Health Organization air quality guideline 2021 interim target 2 (24-hour mean).

^f^ World Health Organization air quality guideline 2005 (24-hour mean).

^g^ Cutoff concentration generated from a study based on data across 18 countries (details in eMethods 3 in Supplement 1).

The city-level urbanization rate appeared to attenuate the associations between ambient air pollutants and hospitalizations for schizophrenia, with seemingly apparent trends shown for PM_10_ (β = −0.0021; SE = 0.00071; *P* = .003) and nitrogen dioxide (β = −0.0031; SE = 0.0012; *P* = .02) (eFigure 3 in Supplement 1). Consistent modifications by nighttime light intensity were also observed (eFigure 4 in Supplement 1). In the stratified analyses based on the mental health service–level factors, a generally decreasing trend in air pollution–related risk of schizophrenia admissions was found along with the increases in the total and population-weighted numbers of psychiatric hospitals, respectively (eFigure 5 in Supplement 1).

### Associations of APINs and Sustained Increase Events With Hospitalizations for Schizophrenia

**Figure 2.**
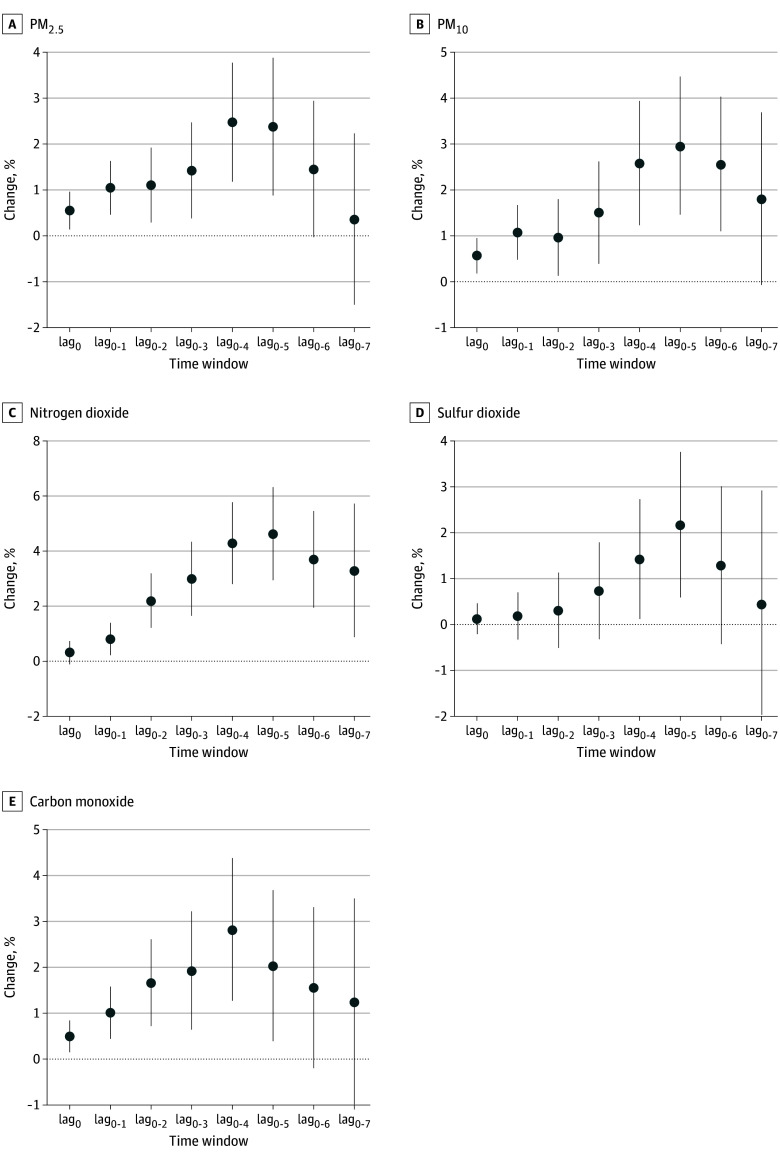
Overall Percentage Changes With 95% CIs in Daily Hospitalizations for Schizophrenia per-IQR Increases in Air Pollution Increases Between Neighboring Days of Different Air Pollutants at Different Time Windows in 259 Chinese Cities, 2013-2017 Lag_0_ indicates current day; lag_0-1_, 2-day moving average of lag_0_ and lag_1_ days; lag_0-2_, 3-day moving average of lag_0_ to lag_2_ days; lag_0-3_, 4-day moving average of lag_0_ to lag_3_ days; lag_0-4_, 5-day moving average of lag_0_ to lag_4_ days; lag_0-5_, 6-day moving average of lag_0_ to lag_5_ days; lag_0-6_, 7-day moving average of lag_0_ to lag_6_ days; and lag_0-7_, 8-day moving average of lag_0_ to lag_7_ days. Error bars indicate 95% CIs. PM_10_ indicates particulate matter with an aerodynamic diameter of 10 μm or less; PM_2.5_, particulate matter with an aerodynamic diameter of 2.5 μm or less.

Figure 2 shows that 5-day or 6-day moving average (lag_0-4 _or lag_0-5_) APINs showed the most apparent associations with schizophrenia hospitalizations. Per-IQR increases in 6-day moving average APINs of PM_2.5_ (18.53 μg/m^3^), PM_10_ (28.43 μg/m^3^), nitrogen dioxide (7.98 μg/m^3^), sulfur dioxide (6.67 μg/m^3^), and carbon monoxide (0.22 mg/m^3^) were associated with increases of 2.37% (95% CI, 0.88%-3.88%; adjusted *P* < .001), 2.95% (95% CI, 1.46%-4.47%; adjusted *P* = .003), 4.61% (95% CI, 2.93%-6.32%; adjusted *P* < .001), 2.16% (95% CI, 0.59%-3.76%; adjusted *P* = .01), and 2.02% (95% CI, 0.39%-3.68%; adjusted *P* = .02) in schizophrenia hospitalizations, respectively. These associations for APINs remained statistically significant after adjusting for the absolute concentrations for respective air pollutants (eTable 6 in Supplement 1).

**Figure 3.**
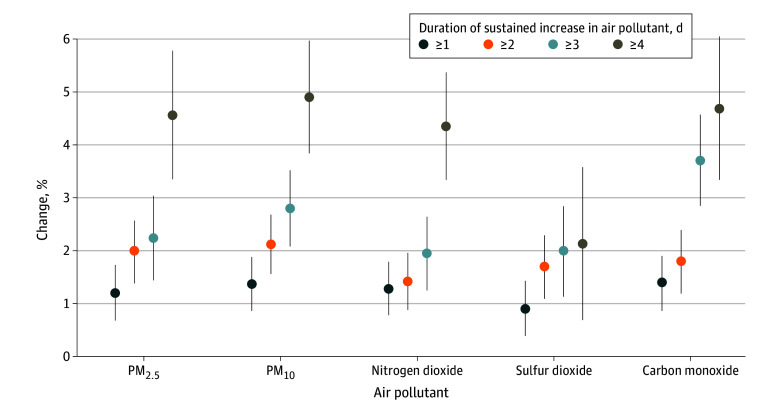
Overall Percentage Changes With 95% CIs in Daily Hospitalizations for Schizophrenia Associated With Sustained Increase Events of Different Ambient Air Pollutants in 259 Chinese Cities, 2013-2017 PM_10_ indicates particulate matter with an aerodynamic diameter of 10 μm or less; PM_2.5_, particulate matter with an aerodynamic diameter of 2.5 μm or less.

Examples of the definitions of sustained increase events in PM_2.5_ for 1, 2, 3, and 4 days are shown in eFigure 6 in Supplement 1. As shown in Figure 3, the associations were more pronounced for sustained increase events with longer durations. The effect estimates for the sustained increase events of different air pollutants at different lag days are provided in eFigure 7 in Supplement 1.

### Associations in the 2-Pollutant Models and Subgroup Analyses

The associations for the absolute concentrations of PM_2.5_, PM_10_, sulfur dioxide, and carbon monoxide were generally attenuated in 2-pollutant models, especially after adjusting for nitrogen dioxide (eTable 7 in Supplement 1). However, the associations for nitrogen oxide remained robust. Similar phenomena were also observed for the associations for APINs in the 2-APIN models (eTable 8 in Supplement 1). In the 2-pollutant models for excess risk due to air pollution concentrations exceeding the WHO-AQGs, the associations for nitrogen dioxide and sulfur dioxide remained generally unchanged (eTable 9 in Supplement 1).

In the subgroup analyses, all the ambient air pollutants consistently showed more obvious associations in male patients than in female patients (eFigure 8 in Supplement 1), although an apparent subgroup difference was only observed for nitrogen dioxide (5.42% [95% CI, 3.48%-7.41%] vs 2.36% [95% CI, 0.75%-4.00%], *P* = .02). More apparent associations were also consistently observed for patients enrolled in the URBMI than those enrolled in the UEBMI, and the subgroup difference was statistically significant for nitrogen dioxide (6.18% [95% CI, 4.02%-8.37%] vs 2.26% [95% CI, 0.40%-4.16%], *P* = .007) and carbon monoxide (3.28% [95% CI, 1.38%-5.22%] vs 0.60% [95% CI, −1.03% to 2.25%], *P* = .04).

A substantial proportion of hospitalizations for schizophrenia was attributable to air pollutants, with the crude attributable fractions ranging from 1.78% (95% CI, 0.68%-2.87%) for PM_2.5_ to 6.22% (95% CI, 4.12%-8.26%) for nitrogen dioxide (eFigure 9 in Supplement 1). The schizophrenia hospitalizations attributable to air pollution concentrations exceeding the WHO-AQGs or specific threshold concentrations were smaller (eFigure 10 and eResults 1 in Supplement 1). In the meta-regression analyses, an inverse modification by annual mean sulfur dioxide concentration on the association between sulfur dioxide and schizophrenia hospitalizations was observed (eTable 10 in Supplement 1). These results were generally robust in sensitivity analyses (eTables 11-13 and eFigures 11 and 12 in Supplement 1), with detailed description in eResults 2 in Supplement 1. Associations for different air pollutants, except for carbon monoxide, remained unchanged after adjusting for the generalized propensity score variable (eTable 14 in Supplement 1).

## Discussion

This nationwide study found positive associations between absolute concentrations of PM_2.5_, PM_10_, nitrogen dioxide, sulfur dioxide, and carbon monoxide and hospitalizations for schizophrenia in the urban population covering all age groups across 259 cities of prefecture-level or above in the Chinese mainland. We introduced a promising exposure indicator reflecting the acute increases in air pollution concentrations, APIN, and found statistically significant associations between APINs of different air pollutants and schizophrenia hospitalizations, independent of the absolute concentrations of respective air pollutants. Particularly, sustained increase events with longer durations for different air pollutants were associated with more pronounced risks of hospitalizations for schizophrenia. These findings suggest that additional attention should be paid to the short-term increases in air pollution levels, especially sustained increases during several days, which may be linked to a greater risk of acute schizophrenia episodes.

We found that, compared with the absolute concentrations of air pollutants, most APINs showed a longer pattern, with more apparent associations with hospitalizations for schizophrenia at later time windows (Figure 1 and Figure 2). Considering that the associations of the absolute concentrations and APINs of air pollutants with schizophrenia hospitalizations were estimated in the same population and the same context of the medical service system, these results suggest that APINs may be more likely to be responsible for the short-term effects of air pollution stimuli on schizophrenia from the biological perspective because there is usually a stress-regulated process for a temporary adaptation after a short-term stressor exposure.^28^ More pronounced associations with schizophrenia hospitalizations observed for sustained increase events with longer durations for air pollutants suggests potential accumulative effects of continuous stress from air pollution increases. It is difficult to directly compare our results with existing studies because no previous studies have investigated the potential adverse effects of short-term and sustained increases in air pollution levels on the risk of acute schizophrenia episodes. Our findings thus provide novel insights for air pollution–related risk of schizophrenia episodes.

As for the associations between absolute air pollution concentrations and hospitalizations for schizophrenia, a previous study^29^ focusing only on PM_2.5_, ozone, and nitrogen dioxide and individuals 65 years or older in the US also found a null association of ozone with hospitalizations for schizophrenia. However, our effect estimate for per–5 μg/m^3^ increase in PM_2.5_ was lower (0.17% [95% CI, 0.07%-0.28%] vs 0.77% [95% CI, 0.11%-1.44%]), and our effect estimate for per–5 ppb increase in nitrogen dioxide was higher (1.87% [95% CI, 1.22%-2.53%] vs 0.64% [95% CI, 0.20%-1.08%]) than the previous study,^29^ which may be due to the heterogeneous population susceptibilities or adaptations in 2 countries, as well as different characteristics of the study populations (general population vs aged population). Ambient median PM_2.5_ level in the US was obviously lower than in China during the respective study periods (9.43 μg/m^3^ in the US vs 38.84 μg/m^3^ in China), but the median nitrogen dioxide level showed an opposite trend (21.25 ppb in the US vs 11.52 ppb in China) in the 2 studies. According to a previous study,^30^ the population residing in areas with good air quality is possibly more vulnerable to an equal unit increase in air pollution levels when compared with the population residing in areas with poor air quality, because residents in areas with poor air quality may have developed tolerance to air pollution during their normal lives, and their susceptibility to air pollution may have been gradually decreased. On the other hand, residents with good air quality would be more susceptible to the occasional high air pollution levels because they always live in a fresh and comfortable air environment. Discussions about the potential biological mechanisms for the associations of short-term increases in air pollution concentrations and schizophrenia episodes are provided in eDiscussion 1 in Supplement 1. Discussions about the potential inverse modification by urbanization rate, the results of subgroup analyses, and the strengths of this study are provided in eDiscussions 2 to 4 in Supplement 1.

### Limitations

This study has several limitations. First, as in most previous studies,^20,31^ the mean air pollution concentration across fixed monitoring stations in each city was used as a proxy for individual exposure, which may result in potential exposure misclassification. However, this exposure misclassification may lead to an underestimation of the effect estimates.^32,33^ From the perspective of AQG formulation, the guideline established is generally regarded as a safe level, and the health alert or target countermeasures for protecting public health are conducted according to the differences between real-time monitoring concentrations from fixed monitoring stations and the guideline levels. Thus, evidence from monitoring station-based studies is probably more appropriate for policymaking and large population-level health risk estimation associated with ambient air pollution. Second, the moderate to high correlations between different air pollutants made it challenging to isolate the independent contributions of different air pollutants, although nitrogen dioxide showed the most robust effect estimates over various analyses. Accordingly, we can only provide the crude attributable fractions of schizophrenia hospitalizations associated with different air pollutants based on the original formula.^34,35^ Third, although hospitalizations are often used as proxies for schizophrenia episodes,^12,13,14^ the hospitalization count in our study likely represents an underestimate of the true number of schizophrenia episodes because a small proportion of individuals who either were not admitted or did not present for treatment cannot be included in the study. Meanwhile, hospitalizations for first-onset and relapse episodes of schizophrenia were not deliberately distinguished due to the unavailability of related information. Considering that acute schizophrenia episodes, both first onset and relapse, may share similar pathological mechanisms, such as immunoinflammatory dysfunction,^36,37,38^ and these adverse pathological changes can be induced or aggravated by air pollution exposure,^39,40^ it may not be necessary to distinguish the potential effects of air pollution on new-onset schizophrenia from preexisting schizophrenia. Fourth, a causal association between the exposure and outcome could not be established due to the study design, but our analysis using the generalized propensity score^41^ may provide evidence to support a potential causal association between air pollution and schizophrenia episodes. Fifth, the study was conducted based on medical insurance schemes for the urban population in the Chinese mainland, and extrapolation of our findings to a wider area (eg, rural areas) may need to be made cautiously. An investigation among the rural population in China based on a relevant medical insurance database is needed in the future to examine the differences in observed associations between urban and rural populations. Because China is a large country with diverse geographic coverage and a wide air pollution concentration range (from very low to very high), our findings may have potential important implications for different parts of the world.

## Conclusions

Our study found that short-term increases in air pollution levels between neighboring days, indicated as APINs, were significantly associated with increased risks of schizophrenia hospitalizations, independent of the absolute concentrations of respective air pollutants. These findings offer novel understanding about air pollution–related risk of schizophrenia episodes and suggest that additional attention should be paid to short-term increases in air pollution levels, especially sustained increases in air pollution levels lasting for longer durations, in the prevention of schizophrenia episodes.

## Notes

Supplement 1. **eMethods 1.** Variables considered in the subgroup and meta-regression analyses **eMethods 2**. Model settings **eMethods 3**. Definitions of excessive or heavily excessive air pollution concentrations **eMethods 4**. Calculation of attributable numbers and attributable fractions for schizophrenia hospitalizations and length of hospital stay associated with short-term exposure to ambient air pollution **eMethods 5**. Calculation of excess numbers of hospitalizations and excess length of hospital stay for schizophrenia due to the excessive or heavily excessive air pollution. **eMethods 6.** Exploratory analyses for the modifications by urbanization and mental health service level. **eMethods 7**. Subgroup and multivariable meta-regression analyses **eMethods 8**. Sensitivity analyses **eResults 1**. Attributable burden of schizophrenia hospitalizations associated with air pollutants and excess burden associated with air pollutants exceeding the WHO-AQGs **eResults 2**. Potential modifications in meta-regression analyses and results for sensitivity analyses **eDiscussion 1**. Potential mechanisms **eDiscussion 2.** The potential inverse modification by urbanization rate **eDiscussion 3**. Subgroup analyses, meta-regression analyses and analyses for attributable fractions **eDiscussion 4**. Strengths of this study **eFigure 1.** Central locations of the 259 Chinese cities of prefecture-level or above included in the study **eFigure 2.** Overall exposure-response curves for the associations between ambient air pollution concentrations at lag 0-1 and daily hospitalizations for schizophrenia in 259 Chinese cities, 2013-2017 **eFigure 3.** Scatter plots of city-specific log relative risks of daily hospitalizations for schizophrenia per IQR increase in concentrations of ambient PM_2.5_ (A), PM_10_ (B), NO_2_ (C)_,_ SO_2_ (D) and CO (E) at lag 0-1 in 259 Chinese cities (Y axis) versus the levels of urbanization (X axis) **eFigure 4.** Scatter plots of city-specific log relative risks of daily hospitalizations for schizophrenia per IQR increase in concentrations of ambient PM_2.5_ (A), PM_10_ (B), NO_2_ (C)_,_ SO_2_ (D) and CO (E) at lag 0-1 in 259 Chinese cities (Y axis) versus the levels of NTLI (X axis) **eFigure 5.** Overall percent changes and 95% confidence intervals in daily hospitalizations for schizophrenia associated with per IQR increase in ambient air pollution concentrations at lag 0-1 in 259 Chinese cities, 2013–2017, classified by the tertiles of city-level total (left panel) and population-weighted (right panel) numbers of psychiatric hospitals **eFigure 6:** Definitions for sustained increase in PM_2.5_ for one, two, three and four days. **eFigure 7.** Overall percent changes with 95% confidence intervals in daily hospitalizations for schizophrenia associated with sustained increase events of air pollutants at different lag days in 259 Chinese cities, 2013-2017 **eFigure 8.** Overall percent changes (%) with 95% confidence intervals in daily hospitalizations for schizophrenia per IQR increase in ambient air pollution concentrations at lag 0-1 in subgroups in 259 Chinese cities, 2013-2017 **eFigure 9.** Attributable numbers and fractions with 95% confidence intervals in daily hospitalizations (A) and length of hospital stay (B) for schizophrenia associated with short-term exposure to ambient air pollutants at lag 0-1 in 259 Chinese cities, 2013-2017 **eFigure 10.** Excess numbers and fractions with 95% confidence intervals in daily hospitalizations (A) and length of hospital stay (B) for schizophrenia associated with excessive or heavily excessive air pollutant concentrations under different definitions in 259 Chinese cities, 2013-2017 **eFigure 11.** Overall percent changes with 95% confidence intervals in daily hospitalizations for schizophrenia per IQR increase in ambient air pollution concentrations at different lag days in 259 Chinese cities after replacing the ambient air pollution data from the China National Environmental Monitoring Centre with data from the CAQRA dataset, 2013-2017 **eFigure 12.** Overall percent changes with 95% confidence intervals in daily hospitalizations for schizophrenia per IQR increase in ambient air pollution concentrations at different lag days in 212 Chinese cities with data from both the UEBMI and URBMI, 2013-2017 **eTable 1.** Intraclass correlation coefficient between ambient air pollution data from the China National Environmental Monitoring Centre and ambient air pollution data from the CAQRA dataset in 259 Chinese cities, 2013-2017 **eTable 2.** Demographic characteristics of patients admitted for schizophrenia in 259 Chinese cities, 2013–2017 **eTable 3.** The 259 Chinese cities of prefecture-level or above included in the study **eTable 4.** Spearman’s correlations between daily ambient air pollutants in 259 Chinese cities, 2013-2017 **eTable 5.** Spearman’s correlations between daily APINs and absolute concentrations of air pollutants in 259 Chinese cities, 2013-2017 **eTable 6.** Overall percent changes with 95% confidence intervals in daily hospitalizations for schizophrenia per IQR increase in APINs at lag 0-5 after adjusting for respective absolute air pollution concentrations in 259 Chinese cities, 2013-2017 **eTable 7.** Overall percent changes with 95% confidence intervals in daily hospitalizations for schizophrenia per IQR increase in ambient air pollution concentrations in two-pollutant models at lag 0-1 in 259 Chinese cities, 2013-2017 **eTable 8.** Overall percent changes with 95% confidence intervals in daily hospitalizations for schizophrenia per IQR increase in APINs in two-APIN models at lag 0-5 in 259 Chinese cities, 2013-2017 **eTable 9.** Overall percent changes with 95% confidence intervals in daily hospitalizations for schizophrenia associated with excessive or heavily excessive air pollution concentrations under different definitions in 259 Chinese cities, 2013-2017 **eTable 10.** Multivariable meta-regression coefficients with 95% confidence intervals of the modifications of city-level characteristics on the associations between ambient air pollution concentrations at lag 0-1 and daily hospitalizations for schizophrenia in 259 Chinese cities, 2013-2017 **eTable 11.** Results of sensitivity analyses on the associations between per IQR increase in ambient air pollution concentrations at lag 0-1 and daily hospitalizations for schizophrenia in 259 Chinese cities, 2013-2017 **eTable 12.** Overall percent changes and 95% confidence intervals in daily hospitalizations for schizophrenia associated with per IQR increase in ambient air pollution concentrations at lag 0-1 in 259 Chinese cities, 2013–2017, classified by the median of city-specific annual average co-pollutants concentrations **eTable 13.** Results of sensitivity analyses for the overall percent changes with 95% confidence intervals in daily hospitalizations for schizophrenia per IQR increase in ambient air pollution concentrations at lag 0-1 in models adjusting for different meteorological factors and extreme meteorological conditions in 259 Chinese cities, 2013-2017 **eTable 14.** Overall percent changes with 95% confidence intervals in daily hospitalizations for schizophrenia per IQR increase in ambient air pollution concentrations adjusting for the generalized propensity score in 259 Chinese cities, 2013-2017

Supplement 2.
Data Sharing Statement
