# Supplementary material for: Ambient Air Pollution and Hospitalizations for Schizophrenia in China
Source: JAMA Netw Open. 2024 Oct 2;7(10):e2436915. doi: 10.1001/jamanetworkopen.2024.36915 (PMC11447564; doi:10.1001/jamanetworkopen.2024.36915)
Supplement: Supplement 1. — eMethods 1. Variables considered in the subgroup and meta-regression analyses eMethods 2. Model settings eMethods 3. Definitions of excessive or heavily excessive air pollution concentrations eMethods 4. Calculation of attributable numbers and attributable fractions for schizophrenia hospitalizations and length of hospital stay associated with short-term exposure to ambient air pollution eMethods 5. Calculation of excess numbers of hospitalizations and excess length of hospital stay for schizophrenia due to the excessive or heavily excessive air pollution. eMethods 6. Exploratory analyses for the modifications by urbanization and mental health service level. eMethods 7. Subgroup and multivariable meta-regression analyses eMethods 8. Sensitivity analyses eResults 1. Attributable burden of schizophrenia hospitalizations associated with air pollutants and excess burden associated with air pollutants exceeding the WHO-AQGs eResults 2. Potential modifications in meta-regression analyses and results for sensitivity analyses eDiscussion 1. Potential mechanisms eDiscussion 2. The potential inverse modification by urbanization rate eDiscussion 3. Subgroup analyses, meta-regression analyses and analyses for attributable fractions eDiscussion 4. Strengths of this study eFigure 1. Central locations of the 259 Chinese cities of prefecture-level or above included in the study eFigure 2. Overall exposure-response curves for the associations between ambient air pollution concentrations at lag 0-1 and daily hospitalizations for schizophrenia in 259 Chinese cities, 2013-2017 eFigure 3. Scatter plots of city-specific log relative risks of daily hospitalizations for schizophrenia per IQR increase in concentrations of ambient PM2.5 (A), PM10 (B), NO2 (C), SO2 (D) and CO (E) at lag 0-1 in 259 Chinese cities (Y axis) versus the levels of urbanization (X axis) eFigure 4. Scatter plots of city-specific log relative risks of daily hospitalizations for schizophrenia per IQR increase in concen [file jamanetwopen-e2436915-s001.pdf]

Supplementary Online Content

Bai L, Jiang Y, Wang K, et al. Ambient air pollution and hospitalizations for schizophrenia in China. *JAMA Netw Open*. 2024;7(10):e2436915. doi:10.1001/jamanetworkopen.2024.36915

eMethods 1. Variables considered in the subgroup and meta-regression analyses
eMethods 2. Model settings
eMethods 3. Definitions of excessive or heavily excessive air pollution concentrations
eMethods 4. Calculation of attributable numbers and attributable fractions for schizophrenia hospitalizations and length of hospital stay associated with short-term exposure to ambient air pollution
eMethods 5. Calculation of excess numbers of hospitalizations and excess length of hospital stay for schizophrenia due to the excessive or heavily excessive air pollution.
eMethods 6. Exploratory analyses for the modifications by urbanization and mental health service level.
eMethods 7. Subgroup and multivariable meta-regression analyses
eMethods 8. Sensitivity analyses
eResults 1. Attributable burden of schizophrenia hospitalizations associated with air pollutants and excess burden associated with air pollutants exceeding the WHO-AQGs
eResults 2. Potential modifications in meta-regression analyses and results for sensitivity analyses
eDiscussion 1. Potential mechanisms
eDiscussion 2. The potential inverse modification by urbanization rate
eDiscussion 3. Subgroup analyses, meta-regression analyses and analyses for attributable fractions
eDiscussion 4. Strengths of this study
eFigure 1. Central locations of the 259 Chinese cities of prefecture-level or above included in the study
eFigure 2. Overall exposure-response curves for the associations between ambient air pollution concentrations at lag 0-1 and daily hospitalizations for schizophrenia in 259 Chinese cities, 2013-2017
eFigure 3. Scatter plots of city-specific log relative risks of hospitalizations for schizophrenia per IQR increase in concentrations of ambient $PM_{2.5}$ (A), PM_{10} (B), NO_2 (C), SO_2 (D) and CO (E) at lag 0-1 in 259 Chinese cities (Y axis) versus the levels of urbanization (X axis)
eFigure 4. Scatter plots of city-specific log relative risks of hospitalizations for schizophrenia per IQR increase in concentrations of ambient $PM_{2.5}$ (A), PM_{10} (B), NO_2 (C), SO_2 (D) and CO (E) at lag 0-1 in 259 Chinese cities (Y axis) versus the levels of NTLI (X axis)
eFigure 5. Overall percent changes and 95% confidence intervals in daily hospitalizations for schizophrenia associated with per IQR increase in ambient air pollution concentrations at lag 0-1 in 259 Chinese cities, 2013–2017, classified by the tertiles of city-level total (left panel) and population-weighted (right panel) numbers of psychiatric hospitals
eFigure 6: Definitions for sustained increase in $PM_{2.5}$ for one, two, three and four days.
eFigure 7. Overall percent changes with 95% confidence intervals in daily hospitalizations for schizophrenia associated with sustained increase events of air pollutants at different lag days in 259 Chinese cities, 2013-2017
eFigure 8. Overall percent changes (%) with 95% confidence intervals in daily hospitalizations for schizophrenia per IQR increase in ambient air pollution concentrations at lag 0-1 in subgroups in 259 Chinese cities, 2013-2017
eFigure 9. Attributable numbers and fractions with 95% confidence intervals in daily hospitalizations (A) and length of hospital stay (B) for schizophrenia associated with short-term exposure to ambient air pollutants at lag 0-1 in 259 Chinese cities, 2013-2017
eFigure 10. Excess numbers and fractions with 95% confidence intervals in daily hospitalizations (A) and length of hospital stay (B) for schizophrenia associated with excessive or heavily excessive air pollutant

© 2024 Bai L et al. *JAMA Network Open.*

concentrations under different definitions in 259 Chinese cities, 2013-2017
eFigure 11. Overall percent changes with 95% confidence intervals in daily hospitalizations for schizophrenia per IQR increase in ambient air pollution concentrations at different lag days in 259 Chinese cities after replacing the ambient air pollution data from the China National Environmental Monitoring Centre with data from the CAQRA dataset, 2013-2017
eFigure 12. Overall percent changes with 95% confidence intervals in daily hospitalizations for schizophrenia per IQR increase in ambient air pollution concentrations at different lag days in 212 Chinese cities with data from both the UEBMI and URBMI, 2013-2017
eTable 1. Intraclass correlation coefficient between ambient air pollution data from the China National Environmental Monitoring Centre and ambient air pollution data from the CAQRA dataset in 259 Chinese cities, 2013-2017
eTable 2. Demographic characteristics of patients admitted for schizophrenia in 259 Chinese cities, 2013–2017
eTable 3. The 259 Chinese cities of prefecture-level or above included in the study
eTable 4. Spearman's correlations between daily ambient air pollutants in 259 Chinese cities, 2013-2017
eTable 5. Spearman's correlations between daily APINs and absolute concentrations of air pollutants in 259 Chinese cities, 2013-2017
eTable 6. Overall percent changes with 95% confidence intervals in daily hospitalizations for schizophrenia per IQR increase in APINs at lag 0-5 after adjusting for respective absolute air pollution concentrations in 259 Chinese cities, 2013-2017
eTable 7. Overall percent changes with 95% confidence intervals in daily hospitalizations for schizophrenia per IQR increase in ambient air pollution concentrations in two-pollutant models at lag 0-1 in 259 Chinese cities, 2013-2017
eTable 8. Overall percent changes with 95% confidence intervals in daily hospitalizations for schizophrenia per IQR increase in APINs in two-APIN models at lag 0-5 in 259 Chinese cities, 2013-2017
eTable 9. Overall percent changes with 95% confidence intervals in daily hospitalizations for schizophrenia associated with excessive or heavily excessive air pollution concentrations under different definitions in 259 Chinese cities, 2013-2017
eTable 10. Multivariable meta-regression coefficients with 95% confidence intervals of the modifications of city-level characteristics on the associations between ambient air pollution concentrations at lag 0-1 and daily hospitalizations for schizophrenia in 259 Chinese cities, 2013-2017
eTable 11. Results of sensitivity analyses on the associations between per IQR increase in ambient air pollution concentrations at lag 0-1 and daily hospitalizations for schizophrenia in 259 Chinese cities, 2013-2017
eTable 12. Overall percent changes and 95% confidence intervals in daily hospitalizations for schizophrenia associated with per IQR increase in ambient air pollution concentrations at lag 0-1 in 259 Chinese cities, 2013–2017, classified by the median of city-specific annual average co-pollutants concentrations
eTable 13. Results of sensitivity analyses for the overall percent changes with 95% confidence intervals in daily hospitalizations for schizophrenia per IQR increase in ambient air pollution concentrations at lag 0-1 in models adjusting for different meteorological factors and extreme meteorological conditions in 259 Chinese cities, 2013-2017
eTable 14. Overall percent changes with 95% confidence intervals in daily hospitalizations for schizophrenia per IQR increase in ambient air pollution concentrations adjusting for the generalized propensity score in 259 Chinese cities, 2013-2017
eReferences.

This supplementary material has been provided by the authors to give readers additional information about their work.

eMethods 1: Variables considered in the subgroup analyses and meta-regression analyses

In the subgroup analyses, information on the ranking of cities' business attractiveness was obtained from the China Business Network[1] to explore the differences between the effect estimates for different levels of urban vitality. This ranking variable was generated based on concentration of commercial resources, city as a hub, urban residents' activity, lifestyle diversity, and future potential[2]. In the multivariable meta-regression analyses exploring the potential modification effects of city-level factors, information on the Normalized Difference Vegetation Index (NDVI), cities' annual average air pollution levels, annual average temperature, and gross domestic product (GDP) per capita were collected. The NDVI is used to measure green space, an important city-level factor associated with both air pollution and schizophrenia [3], and was derived from the Moderate Resolution Imaging Spectroradiometer at 1-kilometer spatial resolution in the present study[4]. The data on GDP per capita and urban population were obtained from the China City Statistical Year Book 2017[5].

eMethods 2: Model settings

In our case-crossover study, based on previous evidence on the acute effect of air pollution on neural damage biomarkers, such as brain-derived neurotrophic factor (a critical biomarker of schizophrenia episodes)[6-8], single-day lags from 0d (current day, lag 0) to 3d (previous 3 days, lag 3) and cumulative lags of 0−1d (2-day moving average of lag 0 and lag 1 days, lag 0-1), 0−2d (3-day moving average of lag 0, lag 1 and lag 2 days, lag 0-2), and 0−3d (4-day moving average of lag 0, lag 1, lag 2 and lag 3 days, lag 0-3) before the hospitalization day were respectively used to obtain the current and lagged effect estimates. To explore the lag structure of APIN, cumulative lags of 0-1d (lag 0-1) to 0-7d (lag 0-7) before the hospitalization day were respectively used, including lag 0. The lag 0-1 of APIN refers to an average of the current day's increase relative to yesterday and yesterday's increase relative to the day before. To control for the non-linear confounding effects of meteorological factors, 21-day moving averages of temperature and relative humidity were included in the model using natural cubic splines, with degrees of freedom (df) of 6 and 3, respectively[9,10]. Meanwhile, an indicator variable was generated for public holidays to account for hospitalization variation associated with holidays.

eMethods 3: Definitions of excessive or heavily excessive air pollution concentrations

Before identifying the potential effects of short-term exposure to air pollution above current air quality standards, excessive and heavily excessive air pollution concentrations were defined according to the WHO air quality guideline (AQG) or specific interim target (IT) levels. Based on the characteristics of ambient air pollution in China, with a higher level of $PM_{2.5}$, WHO-AQG-2021-IT-4 level (24h average of 25 μg/m^3) and AQG-2021-IT-1 level (24h average of 75 μg/m^3) were used to define excessive and heavily excessive $PM_{2.5}$ pollution concentrations, respectively. WHO-AQG-2021 level (24h average of 45 μg/m^3) and AQG-2021-IT-2 level (24h average of 100 μg/m^3) were used to define excessive and heavily excessive PM_{10} pollution concentrations, respectively. AQG-2021 level (24h average of 25 μg/m^3) and AQG-2021-IT-2 level (24h average of 50 μg/m^3) were used to define excessive and heavily excessive NO_2 pollution concentrations, respectively; AQG-2005 (24h average of 20 μg/m^3) and AQG-2021 (24h average of 40 μg/m^3) levels were used to define excessive and heavily excessive SO_2 pollution concentrations, respectively.

In view that the P_{99} of CO concentrations across 259 Chinese cities during 2013-2017 in this study is 3.32 mg/m^3, lower than the limit of WHO AQG (24h average of 4 mg/m^3), the limit of 4 mg/m^3 is probably not sufficient enough to protect public health. In accordance with our findings, previous

© 2024 Bai L et al. *JAMA Network Open.*

multicity studies also found no evidence for a threshold in the association of CO with health risk, suggesting reductions in ambient CO levels, even in cities meeting the current air quality guidelines, could yield important health benefits [11,12]. Emissions of ambient CO are mainly from incomplete combustion of fuels from vehicles, whereas the contribution from natural sources is generally low (e.g., 0.15 mg/m³ in the USA) [13]. A study based on daily mortality data of 337 cities in 18 countries or regions covering various periods from 1979 to 2016 found that when the cutoff value of CO decreased from 4.0 mg/m³ to 1.5 mg/m³, the risk estimates remained similar and consistent with the main analysis, but increased when restricting CO concentrations to less than 1 mg/m³ down to less than 0·6 mg/m³ [12]. Therefore, 0.6 mg/m³ and 1.5 mg/m³ were tentatively used to define the excessive and heavily excessive CO pollution concentrations in the present study.

eMethods 4: Calculation of attributable numbers and attributable fractions for schizophrenia hospitalizations and length of hospital stay associated with short-term exposure to ambient air pollution.

The attributable numbers (ANs) and attributable fractions (AFs) of hospitalizations and length of hospital stay (LOS) for schizophrenia associated with exposure to ambient air pollutants were calculated as follows [14]:

$$AN_i = \frac{e^{\{\log(RR) \times X_i\}} - 1}{e^{\{\log(RR) \times X_i\}}} N_i \quad (1)$$

$$AF_i\% = \frac{\sum_{\{i=1\}}^{m} AN \times F_i}{F_{\{total\}}} \times 100\% \quad (2)$$

where AN_i is the AN of hospitalizations for schizophrenia in city i and AF_i% is the AF of corresponding indicators and city. RR indicates the relative risk of hospitalizations per unit increment in ambient air pollutants exposure. X_i is the annual average concentration of each air pollutant in city i; N_i indicates the number of hospitalizations for schizophrenia in city i; F_i is the per capita LOS in city i; F_{total} is the total number of hospitalizations or LOS for schizophrenia in the included city. When F_{total} represents the total number of hospitalizations, F_i is set to 1.

eMethods 5: Calculation of excess numbers of hospitalizations and excess length of hospital stay for schizophrenia due to the excessive or heavily excessive air pollution.

The excess numbers of hospitalizations (EN) and length of hospital stay (EL) for schizophrenia associated with exposure to excessive or heavily excessive air pollution concentrations were calculated as follows:[15]

$$EN = (RR - 1) \times A_{admissions} \times H_{day} \quad (3)$$

$$EL = (RR - 1) \times A_{admissions} \times H_{day} \times A_{LOS} \quad (4)$$

where EN is excess numbers of hospitalizations; EL is excess LOS; RR is the estimated risk with heavy air pollution concentrations; $A_{admissions}$ is the average number of daily hospitalizations for schizophrenia during the study period; H_{day} is the number of days with excessive and heavily excessive air pollution concentration during the study period; and A_{LOS} is the average LOS for each hospitalization. The excess fractions (EFs) of hospitalizations or LOS were calculated through dividing the corresponding ENs by the total number of hospitalizations or LOS during the study period.

eMethods 6: Exploratory analyses for the modifications by urbanization and mental health service level

© 2024 Bai L et al. *JAMA Network Open.*

Considering that urbanization and mental health service level factors may influence air pollution-related hospitalizations for schizophrenia, scatter plots were drawn to evaluate the variation in city-specific associations vs. the city urbanization level using population urbanization rate (the share of the urban residents in the total resident population of a city) and an emerging night-time light intensity (NTLI) as the indicators, respectively[16]. Stratified analyses by the tertiles of city-specific total and population-weighted numbers of psychiatric hospitals collected from the China City Statistical Year Book were used to explore the potential modification by mental health service level on the associations between ambient air pollution and schizophrenia hospitalizations.

eMethods 7: Subgroup and multivariable meta-regression analyses

Subgroup analyses by sex, age (0-39 years, 40-64 years, ≥ 65 years), geographical region (southern region, northern region), insurance type (UEBMI, URBMI), cities' business attractiveness ranking (first- and second-tier cities, third- to fifth-tier cities) and season (cool season: November to April, warm season: May to October) were conducted. Included cities were divided into the southern and northern regions by the Huai River-Qinling Mountains line, considering the substantially different meteorological features and characteristics of ambient air pollution between the south and north of China [9]. Differences between the effect estimates of different subgroups were tested using the two-sample Z-test [17]. We also evaluated the potential modification by city-level characteristics, including cities' annual average air pollution level, temperature, per capita gross domestic product (GDP), urban population, and Normalized Difference Vegetation Index (NDVI) in meta-regression models.

eMethods 8: Sensitivity analyses

Several sensitivity analyses were conducted to evaluate the robustness of the main results, including fitting two-pollutant models to control for concomitant air pollutant exposure at the same main time window ($PM_{2.5}$ and PM_{10} were not included simultaneously in the model, due to the high correlation coefficient of 0.90 between these two PMs); changing the degrees of freedom (3-6) for temperature and relative humidity, respectively; changing the time windows (7-, 14-, 21-, and 28-day moving average) for temperature and relative humidity; evaluating the associations after replacing the air pollution data from the National Air Pollution Monitoring Centre with data from the high-resolution air quality reanalysis dataset [18], considering the possible heterogeneity of the two datasets; and estimating the associations in cities with data from both the basic medical insurance scheme (UEBMI) and the urban resident-based basic medical insurance scheme (URBMI) (212 cities). Second, to further reveal the potential confounding effects of exposure to concomitant air pollutants, we also conducted a subgroup analysis by dividing cities into two groups (low co-pollutant exposure and high co-pollutant exposure) based on the median of the annual average concentrations of co-pollutants. Third, considering the potential influence of extreme meteorological events on the observed associations, heavy precipitation events, heavy winds, and extreme conditions of high humidity and low visibility were respectively included in the main models. The heavy precipitation event was defined as precipitation of 50 mm or more in a 24-hour period[19]. The heavy wind event was defined as ≥75th percentile of city-specific wind speed. Fog is featured with visibility less than 1 km and the relative humidity is higher than 90%. Haze is generally featured with visibility less than 10 km and relative humidity lower than 80%. Mist is the intermediate state of fog and haze. To control for the extreme condition of high humidity and low visibility, relative humidity was divided into three levels (<80th percentile, 80-89th percentile and ≥90th percentile of city-specific relative humidity). Finally, continuous levels of daily precipitation (relative

© 2024 Bai L et al. *JAMA Network Open.*

humidity was not included in the model adjusting for precipitation because of the high correlation between precipitation and relative humidity) and wind speed were also respectively adjusted for in the models. Considering the potential association of sunshine duration with schizophrenia episodes[20], we further controlled for daily sunlight hours in the models. Fourthly, mean temperature, relative humidity, wind speed and daily sunlight hours were included in the models simultaneously.

To evaluate the causal associations between air pollution exposure and schizophrenia episodes, we attempted to estimate the generalized propensity score (GPS)[21] and included GPS as a continuous covariate in the model in order to balance the distribution of time-varying factors[22]. The propensity score is defined as the probability that a patient was exposed to increased air pollutant of interest conditional on pre-hospitalizations exposure covariates (meteorological factors). Adjusting for the propensity score will allow for an unbiased estimation of the exposure of interest[22]. The GPS was estimated by using extreme gradient boosting machines, with daily air pollutant exposure as the dependent variable and time-varying factors, including relative humidity, mean temperature, precipitation, and wind speed, as the independent variables.

To investigate the independent association of APINs with hospitalizations for schizophrenia, related absolute air pollutant concentration was included in the main models for each APIN. In the analysis for excess risk of schizophrenia hospitalizations due to exceedance of WHO AQG or specific IT for each air pollutant, two-pollutant models were conducted to control for concomitant excessive or heavily excessive air pollutant concentrations at the same time window (excessive variables for $PM_{2.5}$ and PM_{10} were not included simultaneously in the model, due to the high correlation between these two PMs).

eResults 1: **Attributable burden of schizophrenia hospitalizations associated with air pollutants and excess burden associated with air pollutants exceeding the WHO-AQGs**

Based on the effect estimates obtained from the single-pollutant models for different air pollutants at lag 0-1, the total AFs of hospitalizations for schizophrenia ranged from 1.78% (95% CI 0.68%-2.87%) for $PM_{2.5}$ to 6.22% (95% CI 4.12%-8.26%) for NO_2, with the corresponding ANs ranging from 14,585 (95% CI 5,526-23,463) for $PM_{2.5}$ to 50,814 (95% CI 33,694-67,505) for NO_2 (**eFigure 9**). The AFs of LOS for schizophrenia ranged from 1.84% (95% CI 0.70%-2.96%) for $PM_{2.5}$ to 6.36% (95% CI 4.22%-8.45%) for NO_2 (**eFigure 9**). The excess fractions of schizophrenia hospitalizations associated with excessive air pollution levels ranged from 0.87% (95% CI 0.17%-1.57%) for $PM_{2.5}$ to 2.55% (95% CI 1.93%- 3.18%) for NO_2, and the excess fractions for LOS ranged from 0.85% (95% CI 0.16%-1.54%) for $PM_{2.5}$ to 2.53% (95% CI 1.90%-3.15%) for NO_2 (**eFigure 10**).

eResults 2: Potential modifications in meta-regression analyses and results for sensitivity analyses

The results of meta-regression analyses implied no significant effect modification by annual average air pollutant concentration, annual average temperature, GDP per capita, and NDVI on the observed associations for different air pollutants, except that there was a significant negative modification by annual average air pollutant concentration on the association of ambient SO_2 with hospitalizations for schizophrenia (**eTable 10**).

In the sensitivity analyses, no substantial changes in effect estimates were observed for different air pollutants after changing the degrees of freedom (3-6) and time windows for temperature and relative humidity, respectively (**eTable 11**). Similar associations were observed when replacing the air pollution data from the China National Environmental Monitoring Centre with data from the high-

© 2024 Bai L et al. *JAMA Network Open.*

resolution Chinese air quality reanalysis (CAQRA) dataset (**eFigure 11**). The main results were also consistent when we only included cities (n=212) with data from both the UEBMI and URBMI (**eFigure 12**). In the further analyses stratified by the city-level median annual average concentrations of co-pollutants, NO_2 showed the most robust associations with hospitalizations for schizophrenia in different city subgroups (**eTable 12**). Significant associations were also observed after further adjusting for extreme meteorological conditions (such as heavy precipitation event and heavy wind event) and continuous meteorological variables (such as precipitation, wind speed, and daily sunlight hours) in the main models (**eTable 13**). The association for CO was attenuated after additionally controlling for wind speed and daily sunlight hours in the main models simultaneously (**eTable 13**)

© 2024 Bai L et al. *JAMA Network Open.*

eDiscussion 1: Potential mechanisms

Air pollutants can arrive at the central nervous system through multiple and complex pathways, and the brain has been deemed as a target of air pollutants[23]. The observed associations in this study may be explained by the fact that increased inflammation and oxidative stress induced by air pollutants exacerbate schizophrenic symptoms in people with schizophrenia, consequently leading to a required intensive hospital treatment[24-26]. Immune system dysfunction is considered to play an aetiological role in schizophrenia, people with schizophrenia have a baseline level of inflammatory protein alteration throughout the illness, while individuals with an acute schizophrenia episode might have superimposed immune activity with increased concentrations of baseline inflammatory protein (e.g., interleukin-6)[27]. Acute exposure to ambient air pollution is shown to significantly upregulate inflammatory proteins (e.g., interleukin-6) and exacerbate neurotoxicity[28]. Aberrant immune responses may promote the course of schizophrenia through altered neuroplasticity[29]. Another possible pathway through which air pollutants might trigger schizophrenia episode is aberrant brain functional connectivity. Dysfunctional brain functional connectivity has been linked to acute exacerbations of schizophrenia[30-33]. The functional magnetic resonance imaging of healthy adults revealed that individuals has decreased functional connectivity in widespread regions of the default mode network after a brief exposure (2 hours) to diesel exhaust, compared to those exposed to filtered air[34]. That is, the mechanisms for acute effects of air pollution on schizophrenia exacerbation are possibly associated with oxidative stress, inflammation, and functional connectivity. Sustained increase for two or more days in air pollutant concentrations may induce stronger oxidative stress and aberrant brain functional connectivity, consequently leading to an increased risk of acute schizophrenia episode.

eDiscussion 2: The potential inverse modification by urbanization rate

Intriguingly, we found potential inverse modification by urbanization rate on the observed associations between short-term exposure to ambient air pollution and schizophrenia hospitalizations, which is likely opposite to the expected result. In fact, previous studies have observed a positive impact of urbanization on health[35,36]. Although earlier studies considered that urbanization-associated social fragmentation and deprivation may contribute to the incident schizophrenia[37], recent studies found that the urbanization-schizophrenia association is heterogeneous over countries[38,39]. Additionally, our study concentrated on hospitalizations due to acute schizophrenia episodes, which are strongly influenced by city-specific disease management ability. Higher urbanization is generally accompanied by more sophisticated medical and healthcare systems, higher levels of social reciprocity, better infrastructure, and improved access to mental health services[40], which could help improve mental health management and reduce the chance of acute aggravation of mental disorders (e.g., hospitalizations for schizophrenia). Furthermore, our stratified analyses also suggest a decreasing trend in air pollution-related risk of schizophrenia hospitalizations along with the increases in the city-specific total and population-weighted numbers of psychiatric hospitals, implying a possible protective effect of better mental health service level against air pollution-related risk of schizophrenia hospitalization.

eDiscussion 3: Subgroup analyses, meta-regression analyses and analyses for attributable fractions

We observed more apparent associations in the population enrolled in URBMI than those enrolled in the UEBMI beneficiaries, which may be explained by the difference in individual socioeconomic status (SES). The UEBMI covers employees and those retired, and the URBMI covers residents without previous employment and the unemployed in the urban area [41]. Individuals enrolling in URBMI

generally have no fixed personal income, and low income can entail deprivation of basic needs, which can elevate stress and be associated with increased risks of schizophrenia episodes [42]. Additionally, individuals with lower SES are particularly vulnerable to stressors like air pollution, due to lower education levels and awareness of avoiding higher air pollution [43]. We also found significantly higher effect estimates of NO_2 in males than in females, which could be associated with sex difference in chances of exposure to traffic-related air pollution. NO_2 primarily gets in the air from the burning of fuel, a surrogate for traffic-related air pollutants [44]. According to statistics in China in 2017, males accounted for 71.2% of motor vehicle drivers, far more than female drivers (28.8%) [45]. Unlike particle pollution, which can be of concern over large regions, NO_2 levels are appreciably higher in close proximity to pollution sources (e.g., vehicles), and health effects associated with NO_2 are much less likely farther away from pollution sources[46]. Therefore, higher usage of motor vehicles in males may lead to more opportunities of exposure to high levels of NO_2, contributing to a more obvious increase in schizophrenia hospitalizations associated with ambient NO_2.

In addition, our study identified substantial proportions of schizophrenia hospitalizations and LOS attributable to ambient air pollution, and substantial excess risks of schizophrenia hospitalizations and LOS associated with air pollution concentrations above or below (for CO) current WHO-AQGs. These findings indicate that controlling air pollution levels to below current AQGs could reduce a considerable proportion of disease burden of schizophrenia, and stricter standards or regulations for air pollution control are essential for better health protection.

eDiscussion 4: Strengths of this study

This study has several strengths. First, we provide fresh systematic evidence for the associations between short-term exposure to ambient air pollution and increased hospitalizations for schizophrenia based on the nationwide population in the context of urbanization in China. Second, we advanced the further understanding of the potential effects of acute increase in air pollution levels within a short period of time on schizophrenia hospitalizations by generating a new indicator APIN, and the APIN-related schizophrenia hospitalizations were independent of the main effects of related major air pollutants. This finding could convey a new message to the health care providers that additional attention and prevention measures on the acute increase in air pollution could alleviate related mental health hazards. Third, through use of two different indicators reflecting urbanization level, we found consistent inverse modifications of urbanization level on the air pollution-schizophrenia association, providing new insight into mental health effects associated with well-developed urbanization. Finally, we found population residing in areas with lower air pollution levels showed more obviously increased schizophrenia hospitalizations associated with an equal increment in air pollution levels compared with the population residing in areas with higher air pollution levels, suggesting that regional specificity may be considered in future air pollution prevention and control.

© 2024 Bai L et al. *JAMA Network Open.*

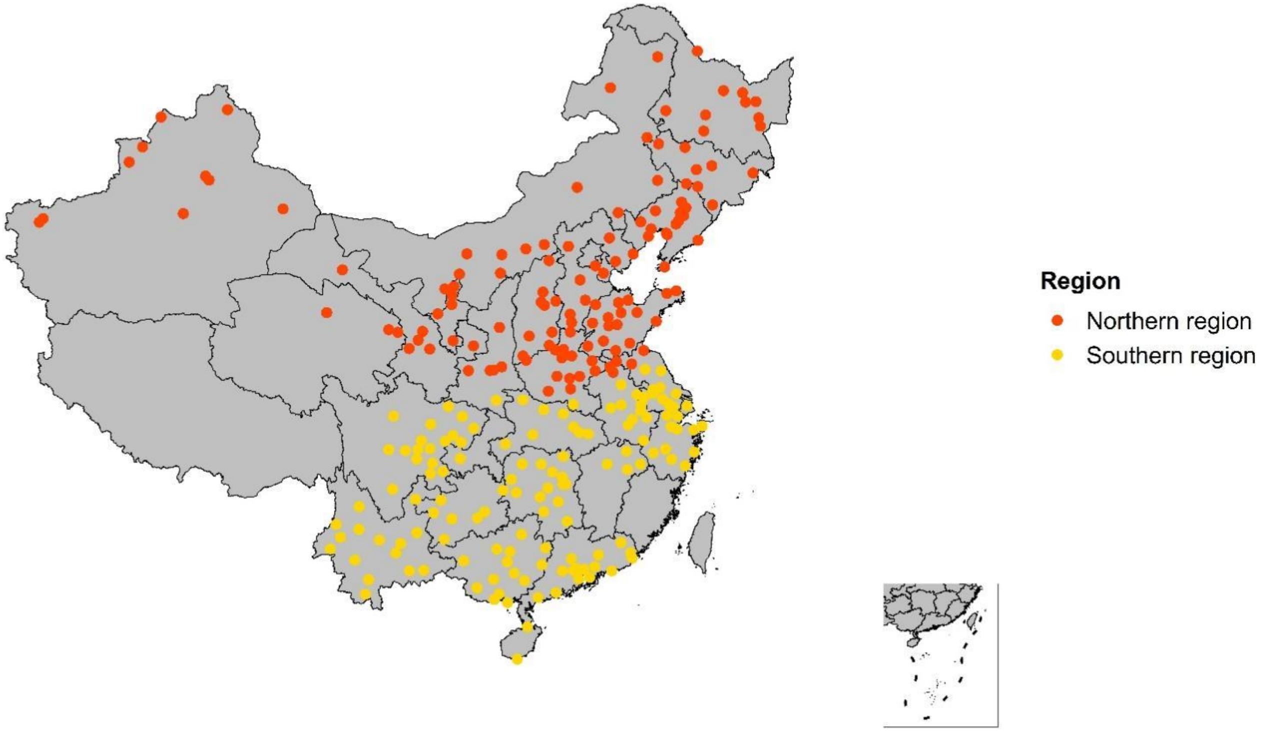

eFigure 1. Central locations of the 259 Chinese cities of prefecture-level or above included in the study

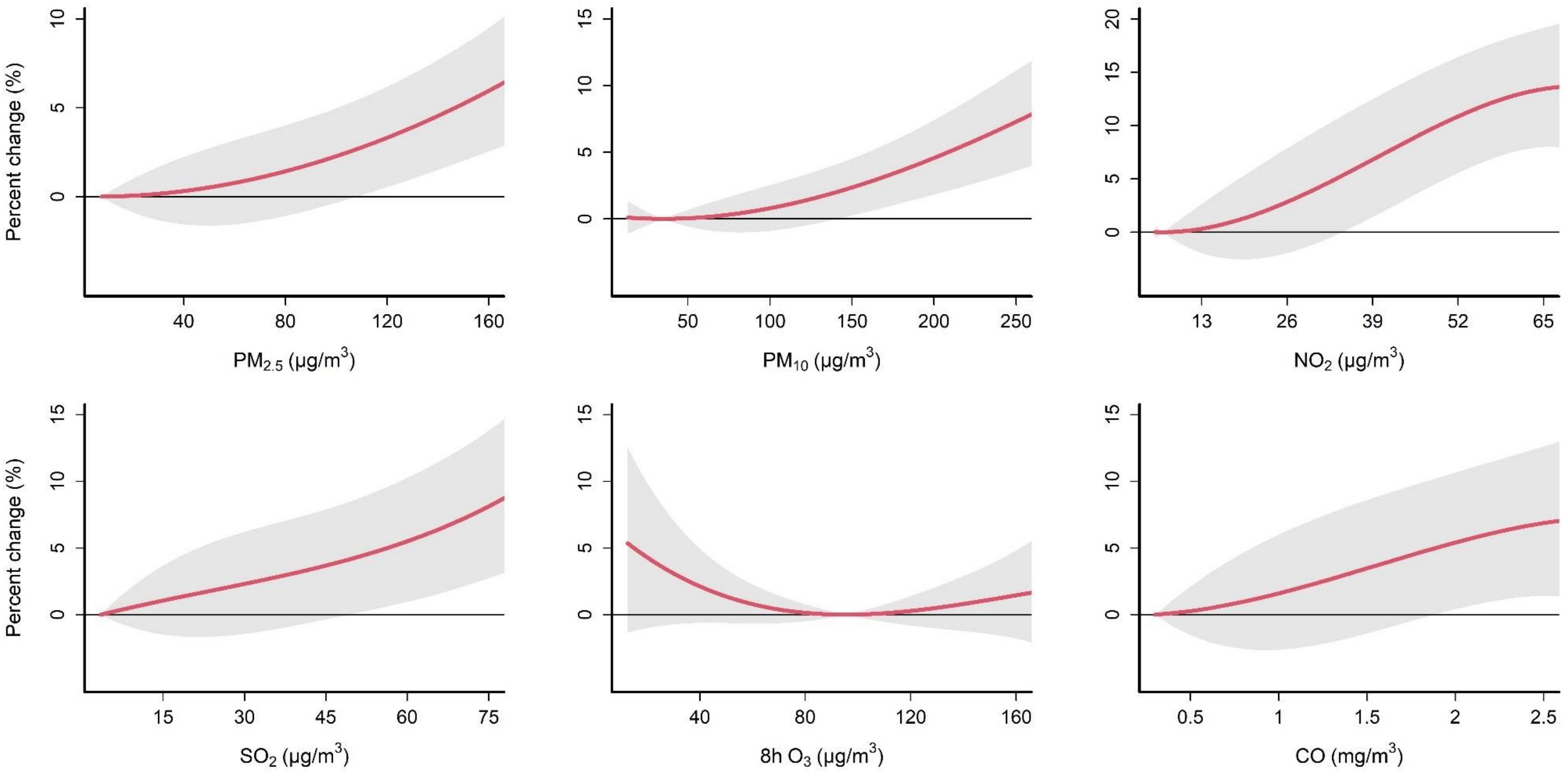

eFigure 2. Overall exposure-response curves for the associations between ambient air pollution concentrations at lag 0-1 and daily hospitalizations for schizophrenia in 259 Chinese cities, 2013-2017.

Abbreviations: CO, carbon monoxide; NO_2, nitrogen dioxide; O_3, ozone; PM_{10}, particulate matter with an aerodynamic diameter of ≤ 10 μm; $PM_{2.5}$, particulate matter with an aerodynamic diameter of ≤ 2.5 μm; SO_2, sulfur dioxide

© 2024 Bai L et al. *JAMA Network Open.*

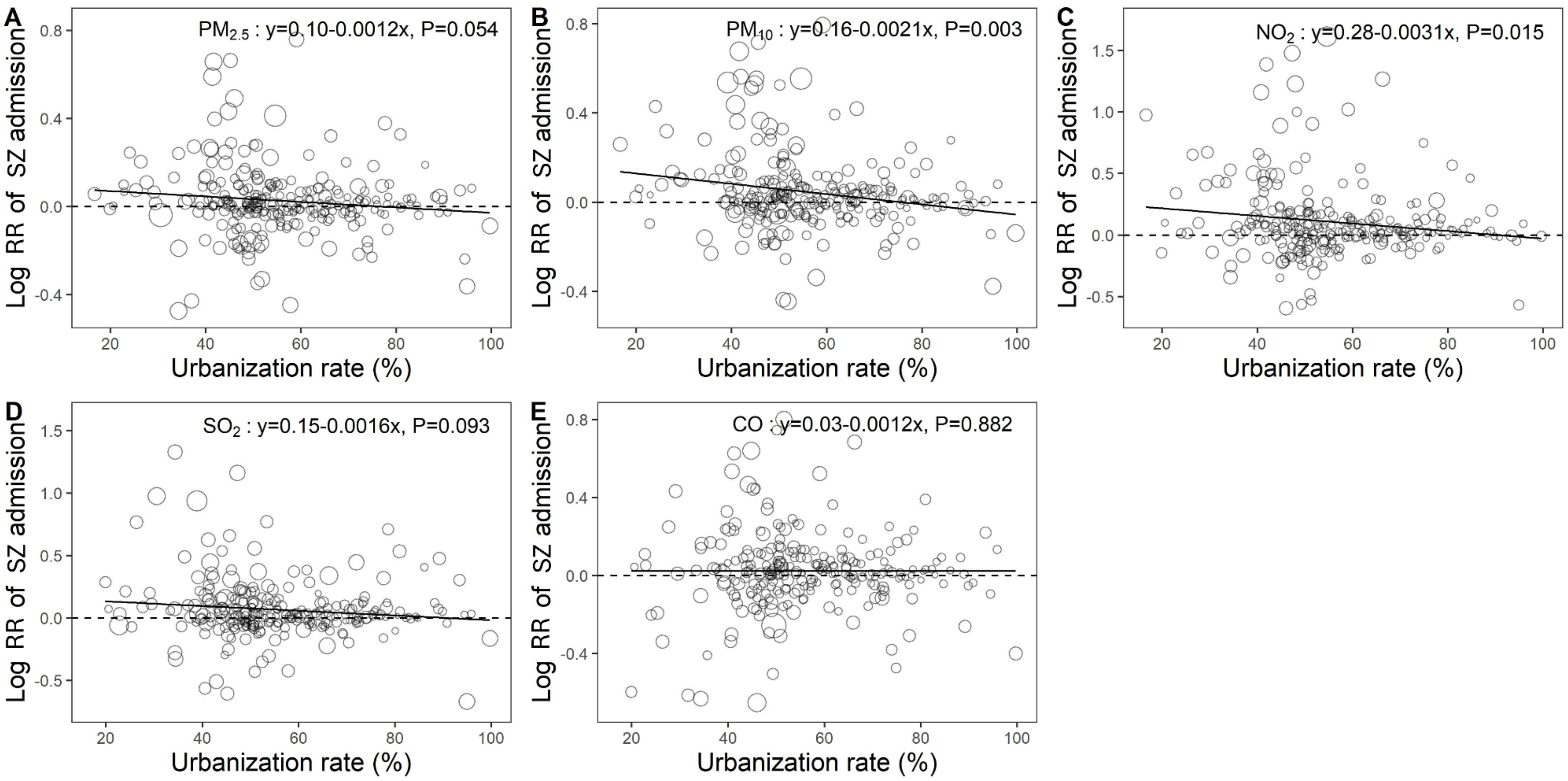

eFigure 3. Scatter plots of city-specific log relative risks of hospitalizations for schizophrenia per IQR increase in concentrations of ambient $PM_{2.5}$ (A), PM_{10} (B), NO_2 (C), SO_2 (D) and CO (E) at lag 0-1 in 259 Chinese cities (Y axis) versus the levels of urbanization (X axis).

Notes: the size of circles is proportional to the standard error of the effect estimate; to control the influence of outliers on linear regression curves, effect estimates lower than the 1st percentile or higher than 99th percentile of all the city-specific effect estimates were excluded in the analysis for each air pollutant.

Abbreviations: CO: carbon monoxide; IQR, interquartile range; NO_2: nitrogen dioxide; RR: rate ratio; PM_{10}: particulate matter with an aerodynamic diameter of ≤ 10μm; $PM_{2.5}$: particulate matter with an aerodynamic diameter of ≤ 2.5μm; SO_2: sulfur dioxide; SZ: schizophrenia.

© 2024 Bai L et al. *JAMA Network Open.*

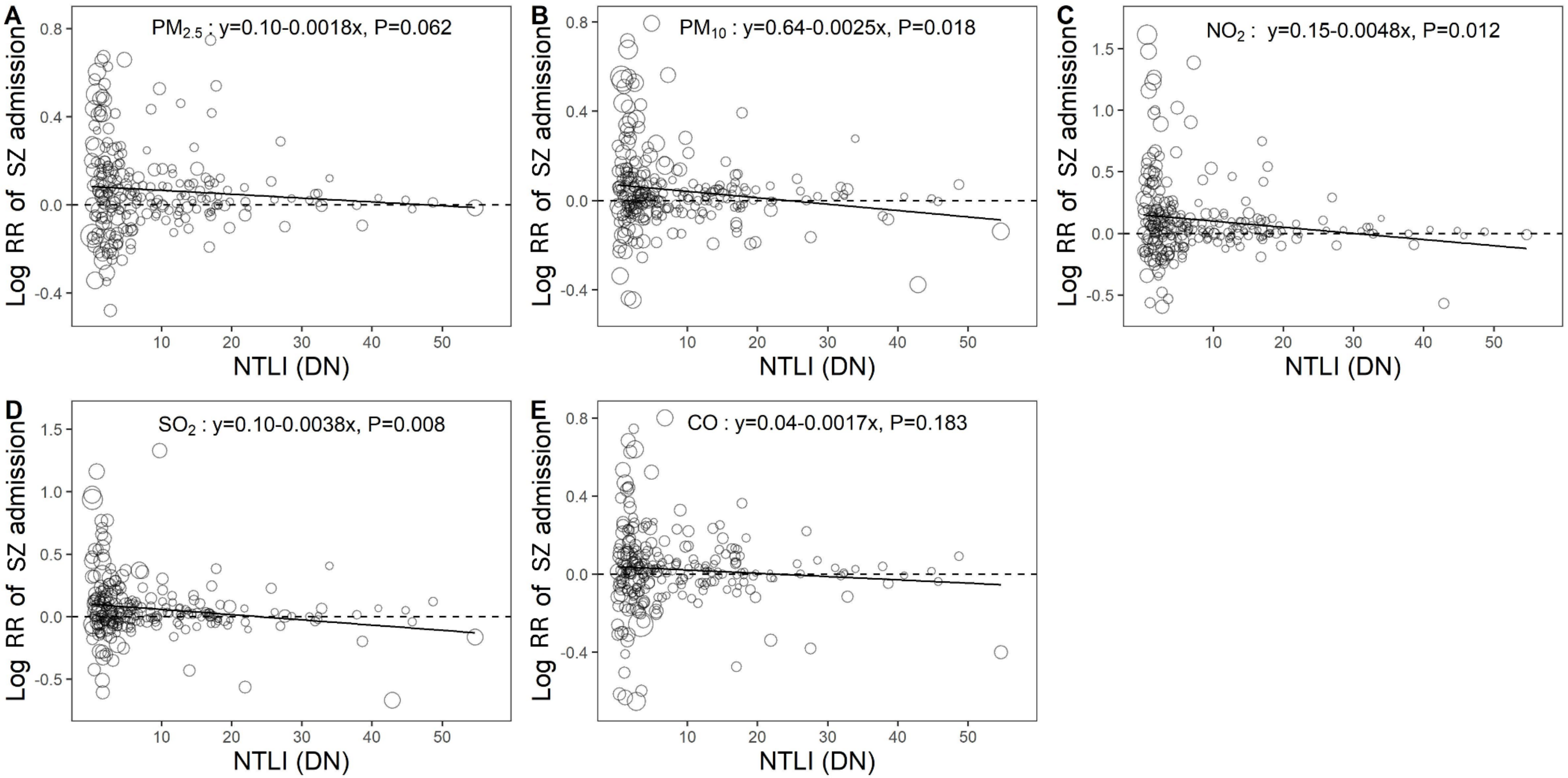

eFigure 4. Scatter plots of city-specific log relative risks of hospitalizations for schizophrenia per IQR increase in concentrations of ambient $PM_{2.5}$ (A), PM_{10} (B), NO_2 (C), SO_2 (D) and CO (E) at lag 0-1 in 259 Chinese cities (Y axis) versus the levels of NTLI (X axis).

Notes: Night-time light intensity (NTLI) is a useful metric for assessing the level of urbanization by identifying urban landscapes, has been deemed as a reliable indicator to assess the level of urbanization [16]; the size of circles is proportional to the standard error of the effect estimate; to control the influence of outliers on linear regression curves, effect estimates lower than the 1st percentile or higher than 99th percentile of all the city-specific effect estimates were excluded in the analysis for each air pollutant.

Abbreviations: CO: carbon monoxide; DN: digital number; IQR, interquartile range; RR: rate ratio; NO_2: nitrogen dioxide; NTLI: night-time light intensity; PM_{10}: particulate matter with an aerodynamic diameter of $\leq$ 10μm; $PM_{2.5}$: particulate matter with an aerodynamic diameter of $\leq$ 2.5μm; SO_2: sulfur dioxide; SZ: schizophrenia.

© 2024 Bai L et al. *JAMA Network Open.*

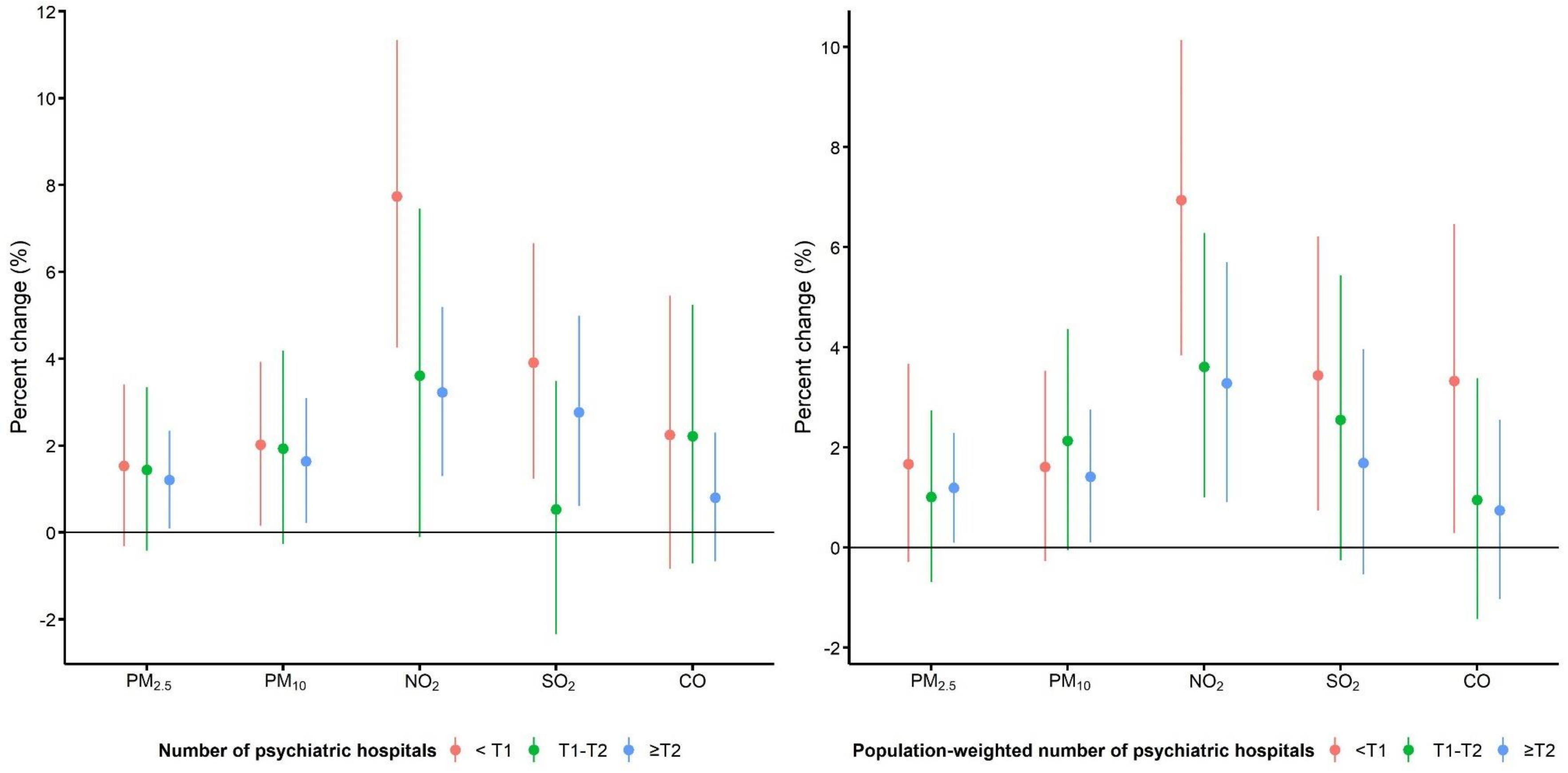

eFigure 5. Overall percent changes and 95% confidence intervals in daily hospitalizations for schizophrenia associated with per IQR increase in ambient air pollution concentrations at lag 0-1 in 259 Chinese cities, 2013–2017, classified by the tertiles of city-level total (left panel) and population-weighted (right panel) numbers of psychiatric hospitals.

Abbreviations: CO: carbon monoxide; IQR, interquartile range; NO_2: nitrogen dioxide; PM_{10}: particulate matter with an aerodynamic diameter of ≤ 10μm; $PM_{2.5}$: particulate matter with an aerodynamic diameter of ≤ 2.5μm; SO_2: sulfur dioxide; T1: the first tertile; T2: the second tertile.

© 2024 Bai L et al. *JAMA Network Open.*

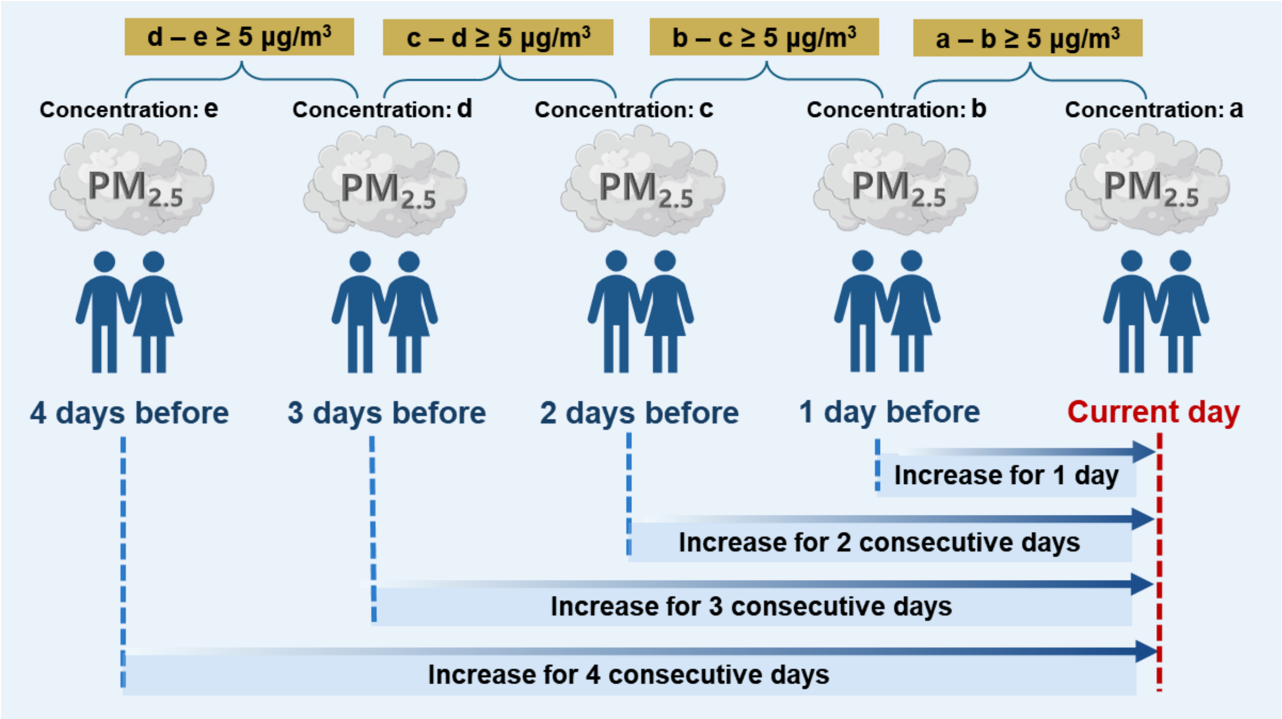

eFigure 6: Definitions for sustained increase in $PM_{2.5}$ for one, two, three and four days.

Abbreviations: $PM_{2.5}$: particulate matter with an aerodynamic diameter of ≤ 2.5μm.

© 2024 Bai L et al. *JAMA Network Open.*

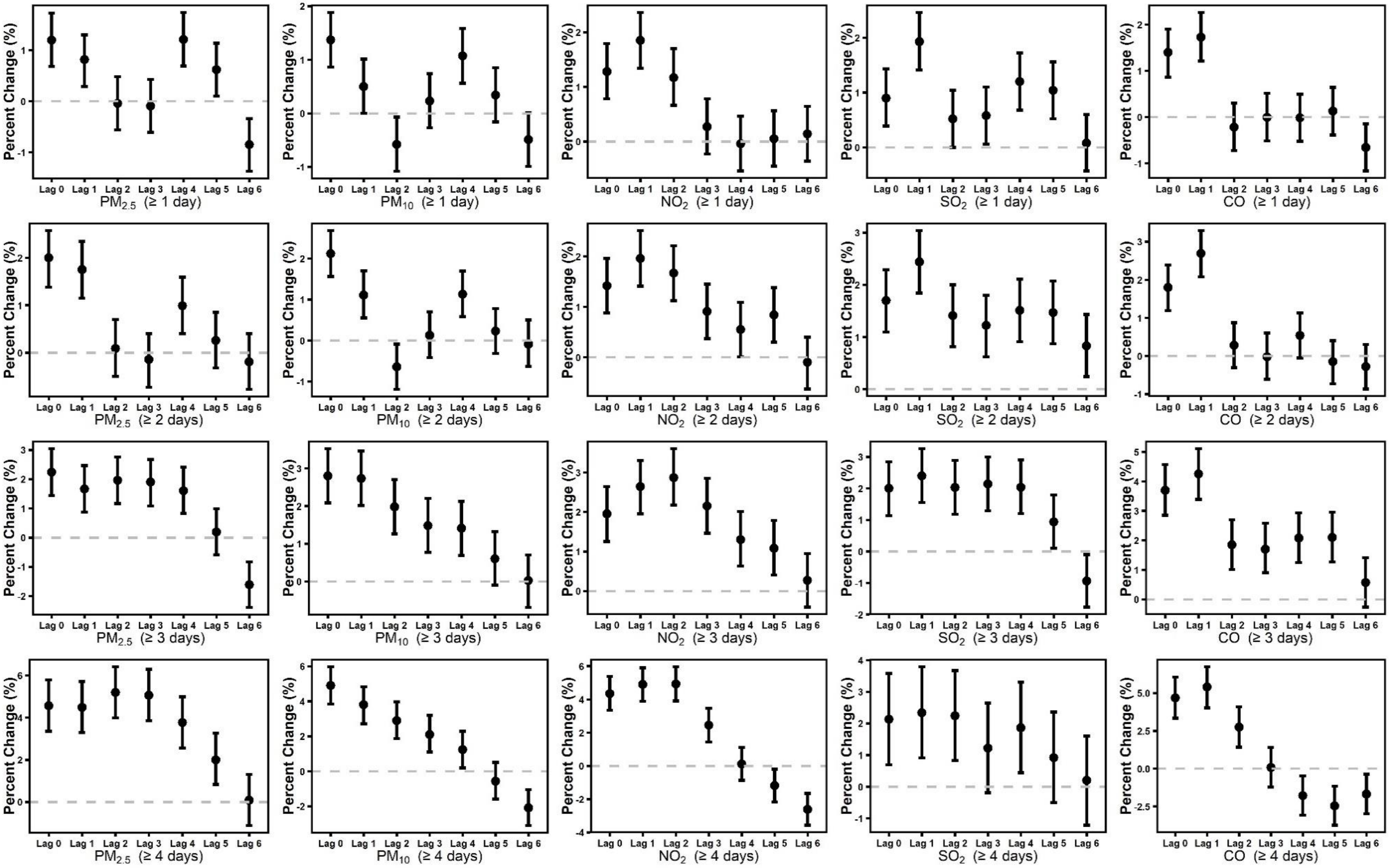

eFigure 7. Overall percent changes with 95% confidence intervals in daily hospitalizations for schizophrenia associated with sustained increase events of air pollutants at different lag days in 259 Chinese cities, 2013-2017.

© 2024 Bai L et al. *JAMA Network Open.*

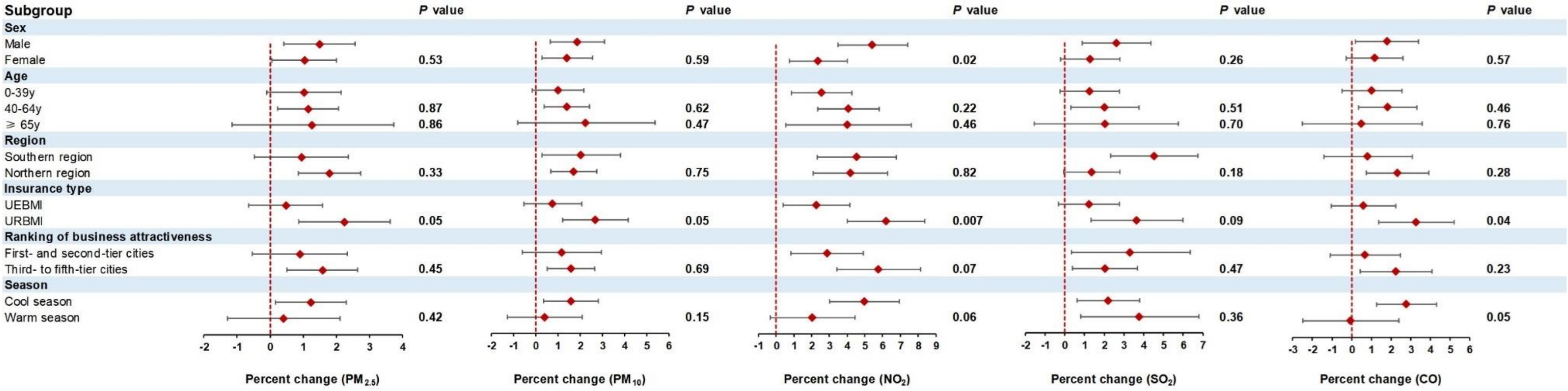

eFigure 8. Overall percent changes (%) with 95% confidence intervals in daily hospitalizations for schizophrenia per IQR increase in ambient air pollution concentrations at lag 0-1 in subgroups in 259 Chinese cities, 2013-2017.

Note: *P* values were derived from a two-sample z-test comparing the differences in the estimates (E) between subgroups (e.g., female vs. male), based on the point estimate and standard error (SE).

Abbreviations: CO: carbon monoxide; IQR, interquartile range; NO_2: nitrogen dioxide; PM_{10}: particulate matter with an aerodynamic diameter of ≤ 10μm; $PM_{2.5}$: particulate matter with an aerodynamic diameter of ≤ 2.5μm; SO_2: sulfur dioxide.

© 2024 Bai L et al. *JAMA Network Open.*

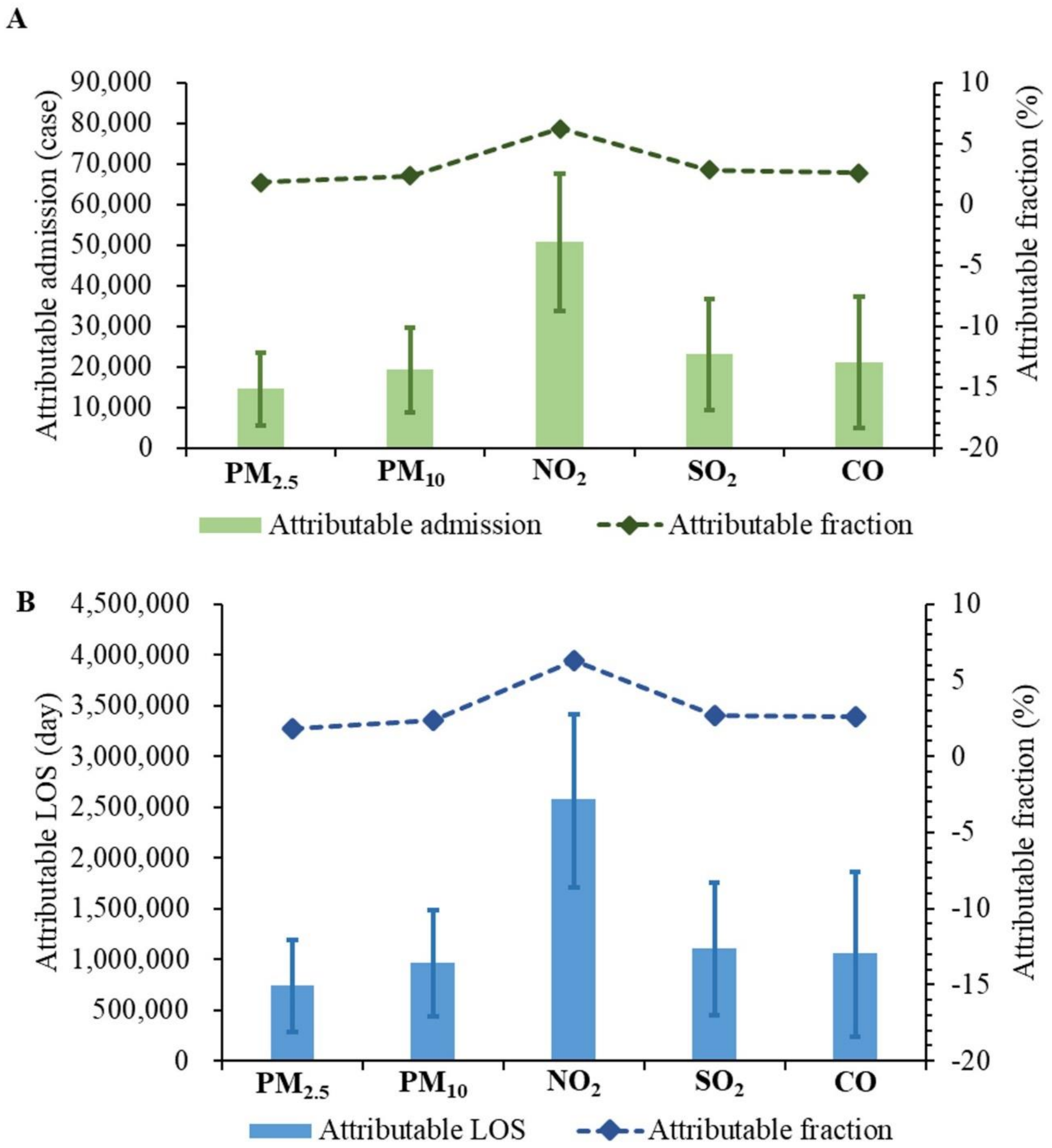

eFigure 9. Attributable numbers and fractions with 95% confidence intervals in daily hospitalizations (A) and length of hospital stay (B) for schizophrenia associated with short-term exposure to ambient air pollutants at lag 0-1 in 259 Chinese cities, 2013-2017.

Abbreviations: CO, carbon monoxide; LOS, length of hospital stay; NO_2, nitrogen dioxide; PM_{10}, particulate matter with an aerodynamic diameter of ≤ 10 μm; $PM_{2.5}$, particulate matter with an aerodynamic diameter of ≤ 2.5 μm; SO_2, sulfur dioxide.

© 2024 Bai L et al. *JAMA Network Open.*

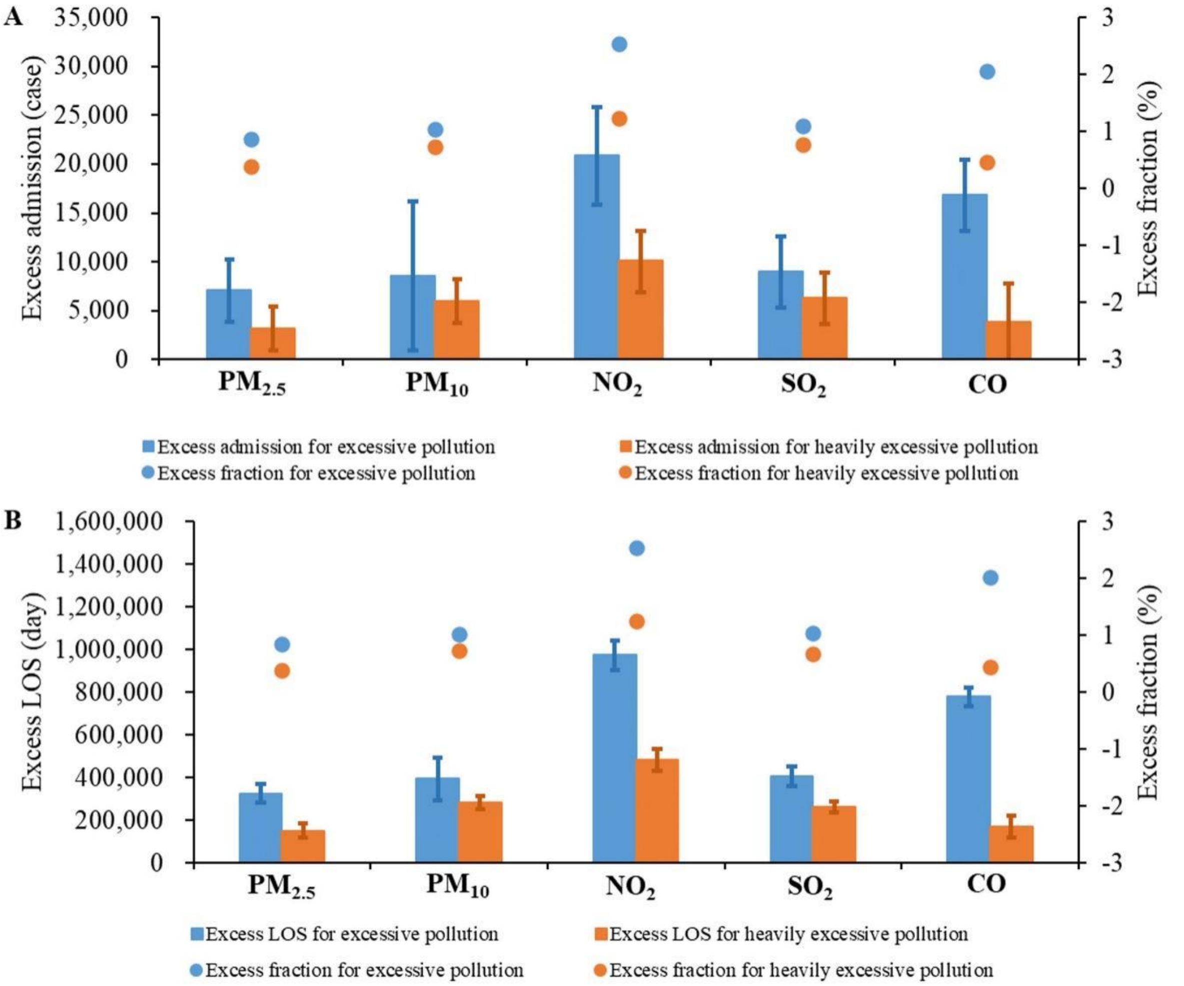

eFigure 10. Excess numbers and fractions with 95% confidence intervals in daily hospitalizations (A) and length of hospital stay (B) for schizophrenia associated with excessive or heavily excessive air pollutant concentrations under different definitions in 259 Chinese cities, 2013-2017.

Note: The excess numbers and fractions of LOS were estimated for 214 cities with available LOS data; excessive and heavily $PM_{2.5}$ concentrations were defined as 25-74 μg/m^3 and ≥ 75 μg/m^3, respectively; excessive and heavily PM_{10} concentrations were defined as 45-99 μg/m^3 and ≥ 100 μg/m^3, respectively; excessive and heavily NO_2 concentrations were defined as 25-49 μg/m^3 and ≥ 50 μg/m^3, respectively; excessive and heavily SO_2 concentrations were defined as 20-39 μg/m^3 and ≥ 40 μg/m^3, respectively; excessive and heavily CO concentrations were defined as 0.6-1.4 mg/m^3 and ≥ 1.5 mg/m^3, respectively.

Abbreviations: CO, carbon monoxide; LOS, length of hospital stay; NO_2, nitrogen dioxide; PM_{10}, particulate matter with an aerodynamic diameter of ≤ 10 μm; $PM_{2.5}$, particulate matter with an aerodynamic diameter of ≤ 2.5 μm; SO_2, sulfur dioxide.

© 2024 Bai L et al. *JAMA Network Open.*

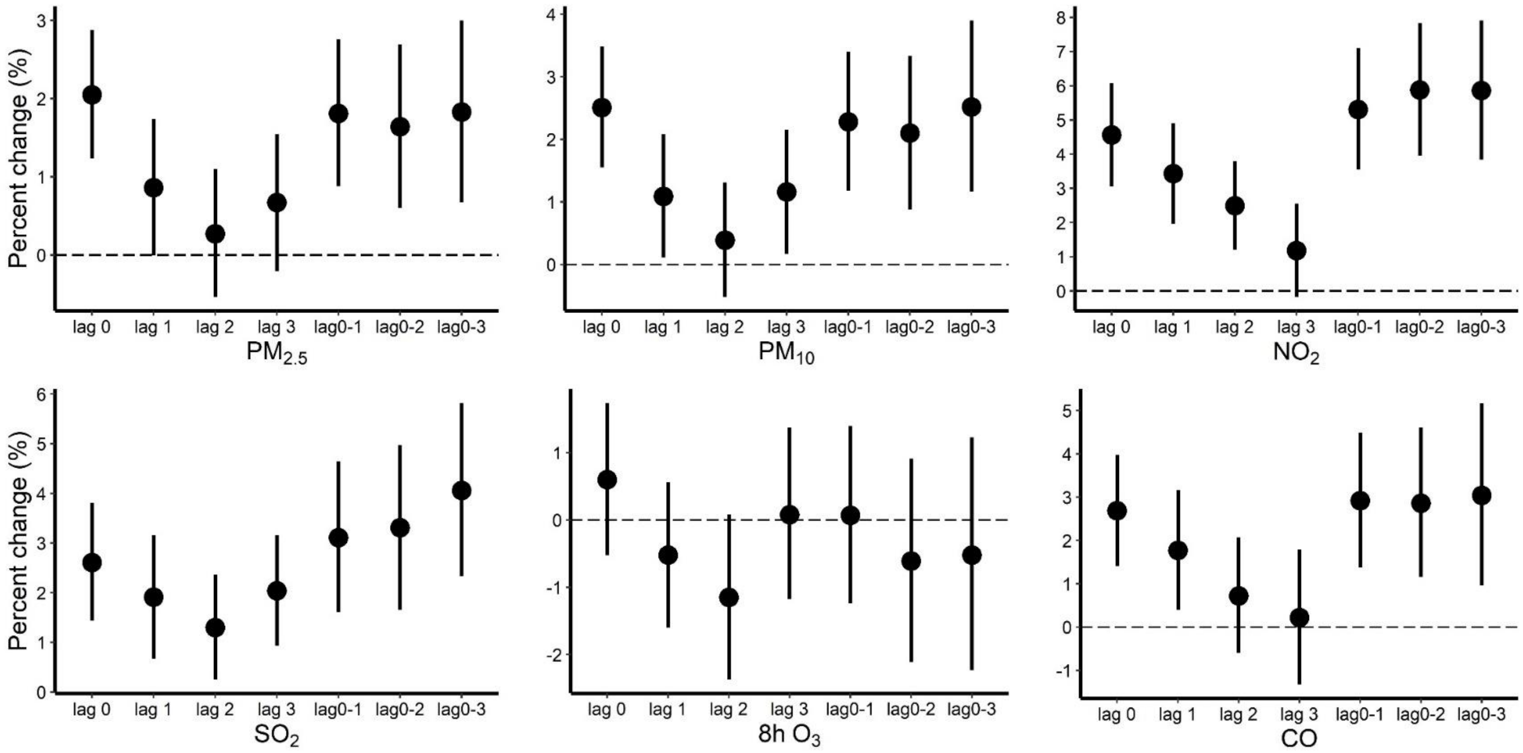

eFigure 11. Overall percent changes with 95% confidence intervals in daily hospitalizations for schizophrenia per IQR increase in ambient air pollution concentrations at different lag days in 259 Chinese cities after replacing the ambient air pollution data from the China National Environmental Monitoring Centre with data from the CAQRA dataset, 2013-2017.

Note: The IQR of each air pollutant across the 259 cities from the CAQRA dataset was 34.68 μg/m^3 for $PM_{2.5}$; 52.97 μg/m^3 for PM_{10}; 19.73 μg/m^3 for NO_2; 13.29 μg/m^3 for SO_2; 42.82 μg/m^3 for 8h O_3; and 0.55 mg/m^3 for CO.

Abbreviations: CAQRA, Chinese air quality reanalysis; CO, carbon monoxide; IQR, interquartile range; NO_2, nitrogen dioxide; O_3, ozone; PM_{10}, particulate matter with an aerodynamic diameter of ≤ 10 μm; $PM_{2.5}$, particulate matter with an aerodynamic diameter of ≤ 2.5 μm; SO_2, sulfur dioxide.

© 2024 Bai L et al. *JAMA Network Open.*

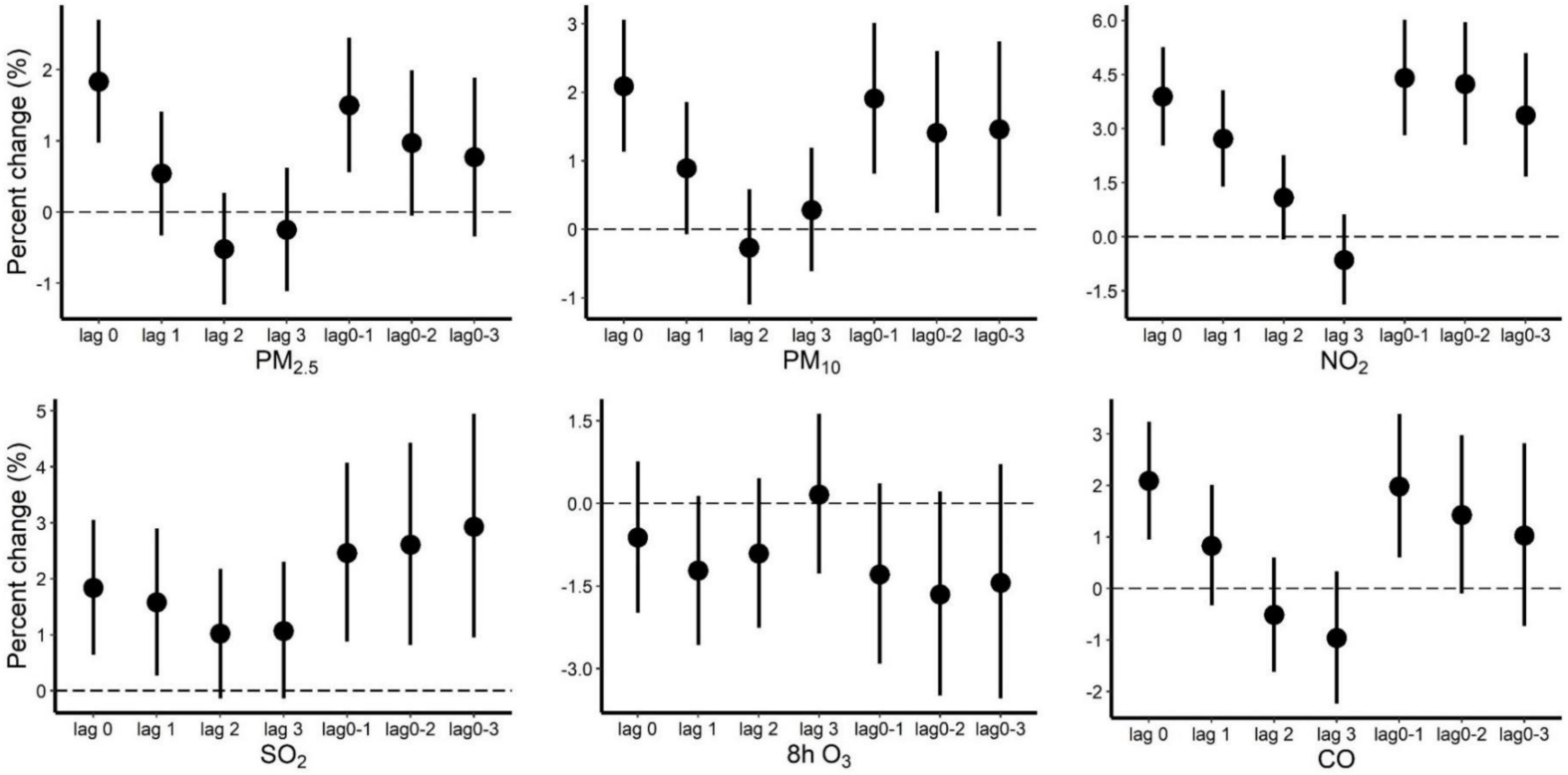

eFigure 12. Overall percent changes with 95% confidence intervals in daily hospitalizations for schizophrenia per IQR increase in ambient air pollution concentrations at different lag days in 212 Chinese cities with data from both the UEBMI and URBMI, 2013-2017.

Abbreviations: CO, carbon monoxide; IQR, interquartile range; NO_2, nitrogen dioxide; PM_{10}, particulate matter with an aerodynamic diameter of ≤ 10 μm; $PM_{2.5}$, particulate matter with an aerodynamic diameter of ≤ 2.5 μm; SO_2, sulfur dioxide. UEBMI, urban employee-based basic medical insurance; URBMI, urban resident-based basic medical insurance.

© 2024 Bai L et al. *JAMA Network Open.*

eTable 1. Intraclass correlation coefficient between ambient air pollution data from the China National Environmental Monitoring Centre and ambient air pollution data from the CAQRA dataset in 259 Chinese cities, 2013-2017.

Air pollutant	ICC and 95%CI
$PM_{2.5}$	0.84 (0.80, 0.86)
PM_{10}	0.74 (0.62, 0.81)
NO_2	0.69 (0.26, 0.85)
SO_2	0.70 (0.57, 0.79)
8h O_3	0.79 (0.75, 0.82)
CO	0.66 (0.49, 0.75)

Abbreviations: CAQRA, Chinese air quality reanalysis; CI, confidence interval; CO, carbon monoxide; ICC, intraclass correlation coefficient; NO_2, nitrogen dioxide; PM_{10}, particulate matter with an aerodynamic diameter of ≤ 10 μm; $PM_{2.5}$, particulate matter with an aerodynamic diameter of ≤ 2.5 μm; SO_2, sulfur dioxide.

© 2024 Bai L et al. *JAMA Network Open.*

eTable 2. Demographic characteristics of patients admitted for schizophrenia in 259 Chinese cities, 2013–2017.

Variable	Overall (N=817 296)	Southern region (N=556 221)	Northern region (N=261 075)
	No (%)	No (%)	No (%)
**Sex**			
Male	449,844 (55.04)	309,904 (55.72)	139,940 (53.60)
Female	367,452 (44.96)	246,317 (44.28)	121,135 (46.40)
**Age (years)**			
< 15	3,584 (0.44)	3,037 (0.55)	547 (0.21)
15-39	246,473 (30.16)	175,546 (31.56)	70,927 (27.17)
40-64	460,988 (56.40)	296,323 (53.27)	164,665 (63.07)
≥ 65	106,251 (13.00)	81,315 (14.62)	24,936 (9.55)
**Insurance type**			
UEBMI	458,289 (56.07)	288,952 (51.95)	169,337 (64.86)
URBMI	359,007 (43.93)	267,269 (48.05)	91,738 (35.14)
**Ranking of business attractiveness** [a]			
First- and second-tier cities	514,562 (62.96)	401,775 (72.23)	112,787 (43.20)
Third- to fifth-tier cities	302,734 (37.04)	154,446 (27.77)	148,288 (56.80)
**Total length of hospital stays (day)**	38,546,114	28,21,6412	10,329,702

Abbreviations: UEBMI, urban employee-based basic medical insurance scheme; URBMI, urban resident-based basic medical insurance scheme.

[a]: The rank of cities' business attractiveness is classified by aggregation index of commercial resource, city hub, activity of urban population, diversity of lifestyle, and flexibility for the future.

© 2024 Bai L et al. *JAMA Network Open.*

eTable 3. The 259 Chinese cities of prefecture-level or above included in the study.

Southern City	Northern City
Aba Tibetan and Qiang Autonomous Prefecture	Altay Prefecture
Ankang	Alxa League
Anshun	Anshan
Baise	Anyang
Baoshan	Baicheng
Bazhong	Baiyin
Beihai	Baoding
Bengbu	Baoji
Bijie	Baotou
Chenzhou	Bayan Nur
Changde	Bayingol Mongolian Autonomous Prefecture
Changsha	Benxi
Changzhou	Binzhou
Chaozhou	Bortala Mongolian Autonomous Prefecture
Chengdu	Bozhou
Chizhou	Changchun
Chongqing	Changji Hui Autonomous Prefecture
Chongzuo	Changzhi
Chuxiong Yi Autonomous Prefecture	Chaoyang
Chuzhou	Chengde
Dali Bai Autonomous Prefecture	Chifeng
Dazhou	Da Hinggan Ling Prefecture
Dehong Dai and Jingpo Autonomous Prefecture	Dalian
Dongguan	Dandong
Enshi Tujia and Miao Autonomous Prefecture	Datong
Fangchenggang	Dezhou
Foshan	Dingxi
Ganzi Tibetan Autonomous Prefecture	Dongying
Guang’an	Fushun
Guangyuan	Fuxin
Guangzhou	Guyuan
Guigang	Harbin
Guilin	Haidong
Haikou	Haixi Mongolian and Tibetan Autonomous Prefecture
Hangzhou	Hami City
Hechi	Handan
Hefei	Hegang
Hengyang	Heihe
Heyuan	Hengshui
Hezhou	Heze
Honghe Hani and Yi Autonomous Prefecture	Hinggan League
Huai’an	Hohhot
Huaihua	Huaibei
Huanggang	Huludao
Huangshan	Hulun Buir
Huizhou	Ili Kazakh Autonomous Prefecture
Huzhou	Jiamusi

Jiaxing
Jingde
Jinhua
Kunming
Laibin
Leshan
Liangshan Yi Autonomous Prefecture
Lijiang
Lincang
Lishui
Liupanshui
Liuzhou
Loudi
Lu’an
Luzhou
Ma’anshan
Maoming
Meishan
Meizhou
Mianyang
Nanchang
Nanchong
Nanjing
Nanning
Nantong
Ningbo
Nujiang Lisu Autonomous Prefecture
Pu’er
Qiandongnan Miao and Dong Autonomous Prefecture
Qiannan Buyi and Miao Autonomous Prefecture
Qianxinan Buyi and Miao Autonomous Prefecture
Qingyuan
Qinzhou
Qujing
Quzhou
Sanya
Shanghai
Shangrao
Shantou
Shanwei
Shaoxing
Shaoyang
Shenzhen
Shiyan
Suining
Suizhou
Suzhou*
Taizhou
Taizhou City

Jiaozuo
Jiayuguan
Jilin
Jinan
Jincheng
Jining
Jinzhong
Jinzhou
Jixi
Kaifeng
Kashgar Prefecture
Kizilsu Kirgiz Autonomous Prefecture
Laiwu
Langfang
Lanzhou
Lianyungang
Liaocheng
Liaoyang
Liaoyaun
Linfen
Linxia Hui Autonomous Prefecture
Linyi
Luohe
Nanyang
Ordos
Panjin
Pingdingshan
Qingdao
Qingyang
Qinhuangdao
Qiqihar
Qitaihe
Sanmenxia
Shangqiu
Shenyang
Shizuishan
Shuangyashan
Siping
Songyuan
Suihua
Suqian
Suzhou
Taian
Taiyuan
Tangshan
Tarbagatay Prefecture
Tianjin
Tieling
Tonghua

© 2024 Bai L et al. *JAMA Network Open.*

Tongling	Tongliao
Tongren	Ulanqab
Wenshan Zhuang and Miao Autonomous Prefecture	Urumqi
Wenzhou	Weifang
Wuhan	Weihai
Wuhu	Weinan
Wuxi	Wuhai
Wuzhou	Wuzhong
Xiangyang	Xi’an
Xiangtan	Xianyang
Xiangxi Tujia and Miao Autonomous Prefecture	Xilingol League
Xiaogan	Xingtai
Xinyang	Xining
Xishuangbanna Dai Autonomous Prefecture	Xinxiang
Xuancheng	Xinzhou
Ya’an	Xuzhou
Yancheng	Yan’an
Yangjiang	Yanbian Chaoxianzu (Korean) Autonomous Prefecture
Yangzhou	Yangquan
Yibin	Yantai
Yichang	Yichun
Yingtan	Yinchuan
Yiyang	Yingkou
Yongzhou	Yuncheng
Yueyang	Zaozhuang
Yulin	Zhangjiakou
Yuxi	Zhengzhou
Zhangjiajie	Zhongwei
Zhaoqing	Zhoukou
Zhaotong	Zhumadian
Zhenjiang	Zibo
Zhongshan	
Zhoushan	
Zhuzhou	
Zigong	
Ziyang	

* There are two cities with the same name in English, this city tagged is from Jiangsu province, China.

Notes: Included cities were divided into the southern and northern regions by the Huai River-Qinling Mountains line.

© 2024 Bai L et al. *JAMA Network Open.*

eTable 4. Spearman's correlations between daily ambient air pollutants in 259 Chinese cities, 2013-2017.

Variable	$PM_{2.5}$	PM_{10}	NO_2	SO_2	8h O_3	CO
$PM_{2.5}$ (μg/m^3)	1.00	0.90	0.60	0.62	-0.03	0.60
PM_{10} (μg/m^3)	..	1.00	0.69	0.66	-0.00	0.64
NO_2 (μg/m^3)	..	..	1.00	0.61	-0.17	0.68
SO_2 (μg/m^3)	..	..	..	1.00	-0.10	0.57
8h O_3 (μg/m^3)	..	..	..	..	1.00	-0.24
CO (mg/m^3)	..	..	..	..	..	1.00

Abbreviations: CO, carbon monoxide; NO_2, nitrogen dioxide; O_3, ozone; PM_{10}, particulate matter with an aerodynamic diameter of ≤ 10 μm; $PM_{2.5}$, particulate matter with an aerodynamic diameter of ≤ 2.5 μm; SO_2, sulfur dioxide.

© 2024 Bai L et al. *JAMA Network Open.*

eTable 5. Spearman's correlations between daily APINs and absolute concentrations of air pollutants in 259 Chinese cities, 2013-2017.

Absolute concentration	APIN of air pollutants	Correlation coefficient
$PM_{2.5}$ (μg/m^3)	APIN of $PM_{2.5}$ (μg/m^3)	0.33
PM_{10} (μg/m^3)	APIN of PM_{10} (μg/m^3)	0.31
NO_2 (μg/m^3)	APIN of NO_2 (μg/m^3)	0.24
SO_2 (μg/m^3)	APIN of SO_2 (μg/m^3)	0.23
8h O_3 (μg/m^3)	APIN of 8h O_3 (μg/m^3)	0.31
CO (mg/m^3)	APIN of CO (mg/m^3)	0.24

Abbreviations: APIN, air pollution increases between neighboring days; CO, carbon monoxide; NO_2, nitrogen dioxide; O_3, ozone; PM_{10}, particulate matter with an aerodynamic diameter of ≤ 10 μm; $PM_{2.5}$, particulate matter with an aerodynamic diameter of ≤ 2.5 μm; SO_2, sulfur dioxide.

© 2024 Bai L et al. *JAMA Network Open.*

eTable 6. Overall percent changes with 95% confidence intervals in daily hospitalizations for schizophrenia per IQR increase in APINs at lag 0-5 after adjusting for respective absolute air pollution concentrations in 259 Chinese cities, 2013-2017.

	**Not adjusting for the absolute air pollutant concentrations**	**Adjusting for the absolute air pollutant concentrations**
APIN for $PM_{2.5}$	2.37 (0.88, 3.88)	2.30 (0.80, 3.82)
APIN for PM_{10}	2.95 (1.46, 4.47)	2.70 (1.23, 4.18)
APIN for NO_2	4.61 (2.93, 6.32)	4.55 (2.86, 6.27)
APIN for SO_2	2.16 (0.59, 3.76)	1.74 (0.19, 3.32)
APIN for CO	2.02 (0.39, 3.68)	2.30 (0.57, 4.05)

Abbreviations: APIN, air pollution increase between neighboring days; CO, carbon monoxide; IQR, interquartile range; NO_2, nitrogen dioxide; PM_{10}, particulate matter with an aerodynamic diameter of ≤ 10 μm; $PM_{2.5}$, particulate matter with an aerodynamic diameter of ≤ 2.5 μm; SO_2, sulfur dioxide.

© 2024 Bai L et al. *JAMA Network Open.*

eTable 7. Overall percent changes with 95% confidence intervals in daily hospitalizations for schizophrenia per IQR increase in ambient air pollution concentrations in two-pollutant models at lag 0-1 in 259 Chinese cities, 2013-2017.

Models	$PM_{2.5}$	PM_{10}	NO_2	SO_2	CO
Single-pollutant model	**1.36 (0.51, 2.21)**	**1.80 (0.81, 2.80)**	**4.32 (2.81, 5.85)**	**2.35 (0.93, 3.79)**	**1.67 (0.37, 2.98)**
Adjusting for $PM_{2.5}$	..	..	**5.25 (3.23, 7.31)**	**2.44 (0.65, 4.26)**	1.40 (-0.41, 3.24)
Adjusting for PM_{10}	..	..	**4.58 (2.61, 6.59)**	**1.87 (0.12, 3.65)**	1.06 (-0.61, 2.70)
Adjusting for NO_2	-0.52 (-1.63, 0.60)	-0.08 (-2.30, 1.15)	..	0.18 (-1.64, 2.04)	-0.49 (-2.26, 1.31)
Adjusting for SO_2	0.68 (-0.42, 1.80)	**1.26 (0.04, 2.50)**	**4.98 (3.04, 6.95)**	..	1.27 (-0.15, 2.72)
Adjusting for CO	0.75 (-0.51, 2.02)	1.28 (-0.01, 2.59)	**5.10 (3.00, 7.24)**	**1.81 (0.18, 3.48)**	..

Abbreviations: CO, carbon monoxide; IQR, interquartile range; NO_2, nitrogen dioxide; PM_{10}, particulate matter with an aerodynamic diameter of ≤ 10 μm; $PM_{2.5}$, particulate matter with an aerodynamic diameter of ≤ 2.5 μm; SO_2, sulfur dioxide.

© 2024 Bai L et al. *JAMA Network Open.*

eTable 8. Overall percent changes with 95% confidence intervals in daily hospitalizations for schizophrenia per IQR increase in APINs in two-APIN models at lag 0-5 in 259 Chinese cities, 2013-2017.

Models	APIN of $PM_{2.5}$	APIN of PM_{10}	APIN of NO_2	APIN of SO_2	APIN of CO
Single-pollutant model	**2.37 (0.88, 3.88)**	**2.95 (1.46, 4.47)**	**4.61 (2.93, 6.32)**	**2.16 (0.59, 3.76)**	**2.02 (0.39, 3.68)**
Adjust for APIN of $PM_{2.5}$	..	..	**4.43 (2.38, 6.52)**	1.35 (-0.52, 3.26)	0.23 (-1.01, 2.32)
Adjust for APIN of PM_{10}	..	..	**3.73 (1.71,5.79)**	0.78 (-1.05, 2.65)	0.09 (-1.83, 2.05)
Adjust for APIN of NO_2	-0.32 (-2.09, 1.49)	0.68 (-1.05, 2.44)	**..**	-0.73 (-2.72, 1.31)	-1.35 (-3.42, 0.77)
Adjust for APIN of SO_2	**1.80 (0.03, 3.61)**	**2.81 (1.06, 4.60)**	**5.09 (2.96, 7.26)**	..	1.24 (-0.65, 3.15)
Adjust for APIN of CO	**1.98 (0.09, 3.91)**	**2.68 (0.92, 4.47)**	**5.68 (3.49, 7.91)**	1.56 (-0.31, 3.47)	..

Abbreviations: APIN, air pollution increases between neighboring days; CO, carbon monoxide; IQR, interquartile range; NO_2, nitrogen dioxide; PM_{10}, particulate matter with an aerodynamic diameter of ≤ 10 μm; $PM_{2.5}$, particulate matter with an aerodynamic diameter of ≤ 2.5 μm; SO_2, sulfur dioxide.

© 2024 Bai L et al. *JAMA Network Open.*

eTable 9. Overall percent changes with 95% confidence intervals [a] in daily hospitalizations for schizophrenia associated with excessive or heavily excessive air pollution concentrations under different definitions in 259 Chinese cities, 2013-2017.

	**$PM_{2.5}$**		**PM_{10}**		**NO_2**		**SO_2**		CO	
	25[b]-74 μg/m³	≥75[c] μg/m³	45[d]-99μg/m³	≥100[e] μg/m³	25[d]-49 μg/m³	≥50[e] μg/m³	20[f]-39 μg/m³	≥40[d] μg/m³	0.6-1.4[g] mg/m³	≥1.5[g] mg/m³
**Single-pollutant model**	**0.88 (0.17, 1.60)**	**1.20 (0.18, 2.20)**	**1.31 (0.60, 2.00)**	**1.54 (0.63, 2.50)**	**3.39 (2.55, 4.23)**	**4.34 (3.24, 5.44)**	**2.45 (1.67, 3.23)**	**3.43 (1.99, 4.90)**	**1.80 (0.86, 2.67)**	**2.30 (0.98, 3.70)**
**Adjusting for $PM_{2.5}$**	..	..	..	..	**3.45 (2.58, 4.34)**	**4.54 (3.33, 6.75)**	**2.42 (1.62, 3.23)**	**3.44 (1.93,4.97)**	**1.55 (0.60, 2.51)**	**2.01 (0.52, 3.52)**
**Adjusting for PM_{10}**	..	..	..	..	**3.32 (2.45, 4.21)**	**4.43 (3.19,5.69)**	**2.37 (1.55, 3.19)**	**3.40 (1.88, 4.94)**	**1.39 (0.46, 2.33)**	**1.84 (0.39, 3.31)**
**Adjusting for NO_2**	-0.09 (-0.83, 0.65)	-0.45 (-1.55, 0.66)	0.41 (-0.32, 3.13)	-0.09 (-1.10, 0.94)	..	..	**1.81 (1.00, 2.62)**	**2.17 (0.66, 3.70)**	0.85 (-0.77, 2.69)	0.78 (-0.65, 2.23)
**Adjusting for SO_2**	0.51 (-0.21, 1.23)	0.18 (-0.87, 1.25)	**0.95 (0.24, 1.67)**	0.53 (-0.43, 1.50)	**3.01 (2.16, 3.87)**	**3.49 (2.33, 4.66)**	..	..	**1.33 (0.42, 2.24)**	1.36 (-0.05, 2.78)
**Adjusting for CO**	0.49 (-0.26, 1.24)	0.58 (-0.52,1.68)	**1.03 (0.30, 1.76)**	**1.07 (0.12, 2.04)**	**3.21 (2.35, 4.08)**	**4.10 (2.95, 5.27)**	**2.28 (1.49, 3.07)**	**3.17 (1.68, 4.68)**	..	..

Abbreviations: CI, confidence intervals; NO_2, nitrogen dioxide; PC, percent changes; PM_{10}, particulate matter with an aerodynamic diameter of ≤ 10 μm; $PM_{2.5}$, particulate matter with an aerodynamic diameter of ≤ 2.5 μm; SO_2, sulfur dioxide.

[a]: The reference category for each air pollutant was the days with low daily air pollution concentrations (< 25 μg/m³ for $PM_{2.5}$; < 45 μg/m³ for PM_{10}, < 25 μg/m³ for NO_2, < 20 μg/m³ for SO_2, and < 0.6 mg/m³ for CO).

[b]: WHO air quality guideline 2021 interim target-4 (24-hour average).

[c]: WHO air quality guideline 2021 interim target-1 (24-hour average).

[d]: WHO air quality guideline 2021 (24-hour average).

[e]: WHO air quality guideline 2021 interim target-2 (24-hour average).

[f]: WHO air quality guideline 2005 (24-hour average).

[g]: Cutoff concentration generated from a study based on data across 18 countries.[12]

© 2024 Bai L et al. *JAMA Network Open.*

eTable 10. Multivariable meta-regression coefficients with 95% confidence intervals of the modifications of city-level characteristics on the associations between ambient air pollution concentrations at lag 0-1 and daily hospitalizations for schizophrenia in 259 Chinese cities, 2013-2017.

	Annual average air pollutant concentration	Annual average temperature (°C)	GDP per capita [a]	Urban population [b]	NDVI [c]
$PM_{2.5}$	-0.0001 (-0.0006, 0.0005)	0.0014 (-0.0010, 0.0038)	-0.0012 (-0.0037, 0.0014)	-0.00002 (-0.0028,0.0027)	-0.0350 (-0.1265, 0.0565)
PM_{10}	-0.0001 (-0.0004, 0.0002)	0.0018 (-0.0011, 0.0047)	-0.0007 (-0.0038, 0.0025)	-0.0001 (-0.0036,0.0034)	-0.0067 (-0.1118, 0.0984)
NO_2	-0.0020 (-0.0040, 0.0001)	0.0034 (-0.0019, 0.0087)	-0.0005 (-0.0065, 0.0055)	-0.0028 (-0.0094, 0.0039)	0.0409 (-0.1640, 0.2457)
SO_2	**-0.0010 (-0.0020, -0.00001)**	0.0019 (-0.0024, 0.0063)	-0.0022 (-0.0067, 0.0023)	0.0026 (-0.0025, 0.0077)	-0.0136 (-0.1704, 0.1431)
CO	-0.0159 (-0.0598, 0.0280)	-0.0007 (-0.0051, 0.0036)	-0.0006 (-0.0054, 0.0042)	-0.0010 (-0.0064, 0.0044)	-0.0354 (-0.1965, 0.1256)

Abbreviations: CO: carbon monoxide; GDP: Gross Domestic Product; IQR, interquartile range; NDVI: Normalized Difference Vegetation Index; NO_2: nitrogen dioxide; PM_{10}: particulate matter with an aerodynamic diameter of ≤ 10μm; $PM_{2.5}$: particulate matter with an aerodynamic diameter of ≤ 2.5μm; SO_2: sulfur dioxide.

[a]: The unit for GDP per capita is ¥ 10000 Yuan.

[b]: The unit for urban population is million person.

[c]: An indicator for city-level residential greenness.

© 2024 Bai L et al. *JAMA Network Open.*

eTable 11. Results of sensitivity analyses on the associations between per IQR increase in ambient air pollution concentrations at lag 0-1 and daily hospitalizations for schizophrenia in 259 Chinese cities, 2013-2017.

	Percent change (95% confidence interval)			
**Change the *df* of mean temperature**				
	df=3	*df*=4	*df*=5	*df*=6 [a]
$PM_{2.5}$	1.27 (0.42, 2.12)	1.31 (0.48, 2.16)	1.36 (0.51, 2.21)	1.36 (0.51, 2.21)
PM_{10}	1.73 (0.74, 2.73)	1.78 (0.79, 2.77)	1.82 (0.83, 2.82)	1.80 (0.81, 2.80)
NO_2	4.28 (2.78, 5.81)	4.28 (2.77, 5.81)	4.26 (2.75, 5.79)	4.32 (2.81, 5.85)
SO_2	2.25 (0.88, 3.65)	2.23 (0.88, 3.60)	2.26 (0.91, 3.63)	2.35 (0.93, 3.79)
CO	1.72 (0.43, 3.03)	1.77 (0.48, 3.08)	1.82 (0.53, 3.13)	1.67 (0.37, 2.98)
**Change the *df* of relative humidity**				
	df=3 [a]	*df*=4	*df*=5	*df*=6
$PM_{2.5}$	1.36 (0.51, 2.21)	1.39 (0.55, 2.23)	1.40 (0.56, 2.24)	1.44 (0.58, 2.81)
PM_{10}	1.80 (0.81, 2.80)	1.81 (0.83, 2.80)	1.82 (0.85, 2.81)	1.88 (0.87, 2.90)
NO_2	4.32 (2.81, 5.85)	4.30 (2.80, 5.82)	4.31 (2.82, 5.83)	4.37 (2.86, 5.9)
SO_2	2.35 (0.93, 3.79)	2.34 (0.92, 3.78)	2.38 (0.95, 3.84)	2.37 (0.94, 3.82)
CO	1.67 (0.37, 2.98)	1.63 (0.35, 2.93)	1.68 (0.39, 2.98)	1.68 (0.39, 2.99)
**Change the *time windows* (moving average) of mean temperature and relative humidity**				
	7-day	14-day	21-day [a]	28-day
$PM_{2.5}$	1.31 (0.43, 2.19)	1.47 (0.61, 2.34)	1.36 (0.51, 2.21)	1.27 (0.42, 2.13)
PM_{10}	1.81(0.81, 2.83)	1.95 (0.95, 2.96)	1.80 (0.81, 2.80)	1.74 (0.76, 2.72)
NO_2	5.42 (3.84, 7.01)	4.95 (3.41, 6.51)	4.32 (2.81, 5.85)	4.25 (2.74, 5.78)
SO_2	2.63 (1.20, 4.09)	2.69 (1.22, 4.17)	2.35 (0.93, 3.79)	2.29 (0.86, 3.73)
CO	1.93 (0.63, 3.25)	1.74 (0.43, 3.06)	1.67 (0.37, 2.98)	1.68 (0.39, 2.99)

Abbreviations: CO, carbon monoxide; *df*, degrees of freedom; NO_2, nitrogen dioxide; PM_{10}, particulate matter with an aerodynamic diameter of ≤ 10 μm; $PM_{2.5}$, particulate matter with an aerodynamic diameter of ≤ 2.5 μm; SO_2, sulfur dioxide.

[a]: Parameters used in the main model.

© 2024 Bai L et al. *JAMA Network Open.*

eTable 12. Overall percent changes and 95% confidence intervals in daily hospitalizations for schizophrenia associated with per IQR increase in ambient air pollution concentrations at lag 0-1 in 259 Chinese cities, 2013–2017, classified by the median of city-specific annual average co-pollutants concentrations.

Co-pollutant	Lower than median	Higher than median
**Percent changes with 95% confidence intervals for $PM_{2.5}$**		
PM_{10}	1.42 (-0.70, 3.58)	1.28 (0.41, 2.16)
NO_2	2.75 (0.60, 4.95)	1.01 (0.11, 1.91)
SO_2	1.91 (0.14, 3.71)	1.19 (0.32, 2.07)
CO	1.52 (0.13, 2.93)	1.26 (0.18, 2.35)
**Percent changes with 95% confidence intervals for PM_{10}**		
$PM_{2.5}$	2.06 (-0.27, 4.44)	1.29 (0.43, 2.15)
NO_2	3.17 (1.00, 5.38)	1.42 (0.32, 2.53)
SO_2	3.01 (1.04, 5.01)	1.29 (0.23, 2.36)
CO	1.82 (0.35, 3.31)	1.73 (0.42, 3.06)
**Percent changes with 95% confidence intervals for NO_2**		
$PM_{2.5}$	4.57 (1.39, 7.85)	4.30 (2.63, 6.00)
PM_{10}	5.56 (2.21, 9.01)	4.06 (2.45, 5.69)
SO_2	5.22 (2.53, 7.98)	3.93 (2.10, 5.79)
CO	7.28 (4.57, 10.07)	2.79 (0.96, 4.66)
**Percent changes with 95% confidence intervals for SO_2**		
$PM_{2.5}$	4.04 (0.83, 7.36)	1.74 (0.21, 3.29)
PM_{10}	5.27 (1.61, 9.05)	1.54 (0.06, 3.04)
NO_2	5.27 (1.61, 9.05)	1.90 (0.38, 3.45)
CO	4.02 (1.68, 6.42)	1.49 (-0.38, 3.41)
**Percent changes with 95% confidence intervals for CO**		
$PM_{2.5}$	0.30 (-2.26, 2.92)	2.44 (0.94, 3.97)
PM_{10}	0.54 (-2.08, 3.23)	2.23 (0.73, 3.75)
NO_2	1.52 (-1.68, 4.84)	1.77 (0.59, 2.97)
SO_2	0.75 (-1.80, 3.36)	2.13 (0.66, 3.63)

Abbreviations: CO, carbon monoxide; IQR, interquartile range; NO_2: nitrogen dioxide; PM_{10}, particulate matter with an aerodynamic diameter of ≤ 10 μm; $PM_{2.5}$, particulate matter with an aerodynamic diameter of ≤ 2.5 μm; SO_2, sulfur dioxide.

eTable 13. Results of sensitivity analyses for the overall percent changes with 95% confidence intervals in daily hospitalizations for schizophrenia per IQR increase in ambient air pollution concentrations at lag 0-1 in models adjusting for different meteorological factors and extreme meteorological conditions in 259 Chinese cities, 2013-2017.

Air pollutant	Main model	Adjusting for heavy precipitation event [a]	Adjusting for heavy wind event [a]	Adjusting for condition of high humidity and low visibility [a]	Adjusting for precipitation [b]	Adjusting for wind speed [b]	Adjusting for daily sunlight hours [b]	Adjusting for wind speed and daily sunlight hours [b]
$PM_{2.5}$	1.36 (0.51, 2.21)	1.31 (0.45, 2.17)	1.17 (0.31, 2.03)	1.13 (0.31, 1.96)	1.28 (0.37, 2.21)	1.21 (0.36, 2.07)	1.17 (0.31, 2.03)	0.90 (0.01, 1.79)
PM_{10}	1.80 (0.81, 2.80)	1.53 (0.53, 2.54)	1.69 (0.69, 2.70)	1.37 (0.43, 2.33)	1.39 (0.33, 2.46)	1.72 (0.73, 2.71)	1.48 (0.51, 2.47)	1.30 (0.28, 2.32)
NO_2	4.32 (2.81, 5.85)	3.72 (2.24, 5.22)	4.32 (2.74, 5.93)	4.48 (2.93, 6.06)	4.26 (2.60, 5.95)	4.37 (2.79, 5.97)	4.52 (2.96, 6.11)	4.34 (2.61, 6.09)
SO_2	2.35 (0.93, 3.79)	1.70 (0.25, 3.17)	2.25 (0.81, 3.71)	2.00 (0.58, 3.45)	1.84 (0.34, 3.36)	2.27 (0.83, 3.73)	2.07 (0.71, 3.45)	1.78 (0.35, 3.24)
CO	1.67 (0.37, 2.98)	1.65 (0.25, 3.08)	1.33 (0.04, 2.63)	1.73 (0.48, 3.00)	2.05 (0.63, 3.49)	1.37 (0.09, 2.67)	1.85 (0.55, 3.16)	1.33 (-0.01, 2.69)

Abbreviations: CO, carbon monoxide; IQR, interquartile range; NO_2, nitrogen dioxide; PM_{10}, particulate matter with an aerodynamic diameter of ≤ 10 µm; $PM_{2.5}$, particulate matter with an aerodynamic diameter of ≤ 2.5 µm; SO_2, sulfur dioxide.

[a]: The specified meteorological factor(s) was included in the model as a categorical variable.

[b] The specified meteorological factor(s) was included in the model as a continuous variable.

Notes: all the meteorological factors in the table were respectively added to the main model that has already adjusted for temperature and relative humidity; the heavy precipitation event was defined as precipitation of 50 mm or more within a 24-hour period; the heavy wind event was defined as $\geq 75^{th}$ percentile of city-specific wind speed; < 80th percentile, 80-89th percentile and $\geq 90^{th}$ percentile of city-specific relative humidity was used to classify different conditions of high humidity and low visibility, with the continuous variable of relative humidity adjusted in the model simultaneously; continuous variable of precipitation and relative humidity were not simultaneously included in the model because precipitation is generally accompanied by high humidity; time window of precipitation, wind speed and daily sunlight hours were same to the main time window of air pollutants (lag 0-1).

© 2024 Bai L et al. *JAMA Network Open.*

eTable 14. Overall percent changes with 95% confidence intervals in daily hospitalizations for schizophrenia per IQR increase in ambient air pollution concentrations adjusting for the generalized propensity score[a] in 259 Chinese cities, 2013-2017.

Air pollutant	Percent change (%) and 95% confidence interval
$PM_{2.5}$	1.27 (0.03, 2.52)
PM_{10}	1.55 (0.15, 2.98)
NO_2	5.57 (3.28, 7.91)
SO_2	1.93 (0.12, 3.77)
CO	1.42 (-0.39, 3.25)

Abbreviations: CO, carbon monoxide; IQR, interquartile range; NO_2, nitrogen dioxide; PM_{10}, particulate matter with an aerodynamic diameter of ≤ 10 μm; $PM_{2.5}$, particulate matter with an aerodynamic diameter of ≤ 2.5 μm; SO_2, sulfur dioxide.

[a]: Generalized propensity score was adjusted as a continuous variable.

© 2024 Bai L et al. *JAMA Network Open.*

eReferences

1. *Ranking of Chinese Cities' Business Attractiveness 2017.* First-tier Cities Research Institute of China Business Network; 2017-05-25 2017.
2. Yicai Global. Ranking of Cities' Business Attractiveness in China 2023. In: The Rising Lab, the city-oriented big data platform of Yicai Media Group.
3. Jimenez RB, Lane KJ, Hutyra LR, Fabian MP. Spatial resolution of Normalized Difference Vegetation Index and greenness exposure misclassification in an urban cohort. *J Expo Sci Environ Epidemiol.* 2022;32(2):213-222.
4. Kamel Didan - University of Arizona, Alfredo Huete - University of Technology Sydney and MODAPS SIPS - NASA. MOD13A3 MODIS/Terra Vegetation Indices Monthly L3 Global 1km SIN Grid. NASA LP DAAC.2015. http://doi.org/10.5067/MODIS/MOD13A3.006.
5. National Bureau of Statistics. China City Statistical Year Book 2017 China National Knowledge Infrastructure. https://data.cnki.net/yearBook/single?id=N2018050234. Published 2018. Accessed.
6. Peng S, Li W, Lv L, Zhang Z, Zhan X. BDNF as a biomarker in diagnosis and evaluation of treatment for schizophrenia and depression. *Discov Med.* 2018;26(143):127-136.
7. Harb M, Jagusch J, Durairaja A, Endres T, Leßmann V, Fendt M. BDNF haploinsufficiency induces behavioral endophenotypes of schizophrenia in male mice that are rescued by enriched environment. *Transl Psychiatry.* 2021;11(1):233.
8. Song JA-O, Qu R, Sun B, et al. Associations of Short-Term Exposure to Fine Particulate Matter with Neural Damage Biomarkers: A Panel Study of Healthy Retired Adults. *Environ Sci Technol.* 2022;56(11):7203-7213.
9. Tian Y, Liu H, Wu Y, et al. Association between ambient fine particulate pollution and hospital admissions for cause specific cardiovascular disease: time series study in 184 major Chinese cities. *BMJ.* 2019;367:l6572.
10. Rai M, Breitner S, Wolf K, Peters A, Schneider A, Chen K. Future temperature-related mortality considering physiological and socioeconomic adaptation: a modelling framework. *Lancet Planet Health.* 2022;6(10):e784-e792.
11. Liu C, Yin P, Chen R, et al. Ambient carbon monoxide and cardiovascular mortality: a nationwide time-series analysis in 272 cities in China. *Lancet Planet Health.* 2018;2(1):e12-e18.
12. Chen K, Breitner S, Wolf K, et al. Ambient carbon monoxide and daily mortality: a global time-series study in 337 cities. *Lancet Planet Health.* 2021;5(4):e191-e199.
13. EPA. *Integrated Science Assessment (ISA) for carbon monoxide.* Washington, DC: U.S. Environmental Protection Agency;2010.
14. Chen G, Zhang Y, Zhang W, et al. Attributable risks of emergency hospital visits due to air pollutants in China: A multi-city study. *Environ Pollut.* 2017;228:43-49.
15. Wei Y, Wang Y, Di Q, et al. Short term exposure to fine particulate matter and hospital admission risks and costs in the Medicare population: time stratified, case crossover study. *BMJ.* 2019;367:l6258.
16. Khan JR, Islam MM, Faisal ASM, Islam H, Bakar KS. Quantification of Urbanization Using Night-Time Light Intensity in Relation to Women's Overnutrition in Bangladesh. *J Urban Health.* 2023;100(3):562-571.
17. Altman DG, Bland JM. Interaction revisited: the difference between two estimates. *Bmj.* 2003;326(7382):219.
18. Xiao Tang LK, Jiang Zhu, et al. A High-resolution Air Quality Reanalysis Dataset over China (CAQRA)[DS/OL]. *Science Data Bank.* 2021.
19. Benestad RE, Parding KM, Erlandsen HB, Mezghani A. A simple equation to study changes in rainfall statistics. *Environmental Research Letters.* 2019;14(8):084017.
20. Liu L, Wu Q, Li X, et al. Sunshine duration and risks of schizophrenia hospitalizations in main urban area: Do built environments modify the association? *Sci Total Environ.* 2023;871:162057.
21. Wu X, Mealli F, Kioumourtzoglou M-A, Dominici F, Braun D. Matching on Generalized Propensity Scores with Continuous Exposures. *J Am Stat Assoc.* 2024;119(545):757-772.
22. Heinze G, Jüni P. An overview of the objectives of and the approaches to propensity score analyses. *Eur Heart J.* 2011;32(14):1704-1708.
23. Genc S, Zadeoglulari Z, Fuss SH, Genc K. The adverse effects of air pollution on the nervous system. *J Toxicol.* 2012;2012:782462.
24. Qiu X, Danesh-Yazdi M, Wei Y, et al. Associations of short-term exposure to air pollution and increased ambient temperature with psychiatric hospital admissions in older adults in the USA: a case-crossover study. *Lancet Planet Health.* 2022;6(4):e331-e341.
25. Flatow J, Buckley P, Miller BJ. Meta-analysis of oxidative stress in schizophrenia. *Biol*

© 2024 Bai L et al. *JAMA Network Open.*

Psychiatry. 2013;74(6):400-409.
26. Wei Q, Ji Y, Gao H, et al. Oxidative stress-mediated particulate matter affects the risk of relapse in schizophrenia patients: Air purification intervention-based panel study. *Environ Pollut.* 2022;292(Pt B):118348.
27. Halstead S, Siskind D, Amft M, et al. Alteration patterns of peripheral concentrations of cytokines and associated inflammatory proteins in acute and chronic stages of schizophrenia: a systematic review and network meta-analysis. *Lancet Psychiatry.* 2023;10(4):260-271.
28. Pradhan SH, Gibb M, Kramer AT, Sayes CM. Peripheral (lung-to-brain) exposure to diesel particulate matter induces oxidative stress and increased markers for systemic inflammation. *Environ Res.* 2023;231(Pt 3):116267.
29. De Picker LJ, Morrens M, Chance SA, Boche D. Microglia and Brain Plasticity in Acute Psychosis and Schizophrenia Illness Course: A Meta-Review. *Front Psychiatry.* 2017;8:238.
30. Higashima M, Takeda T, Kikuchi M, Nagasawa T, Koshino Y. Functional connectivity between hemispheres and schizophrenic symptoms: a longitudinal study of interhemispheric EEG coherence in patients with acute exacerbations of schizophrenia. *Clin EEG Neurosci.* 2006;37(1):10-15.
31. Rubio JM, Lencz T, Barber A, et al. Striatal functional connectivity in psychosis relapse: A hypothesis generating study. *Schizophr Res.* 2022;243:342-348.
32. Mingoia G, Wagner G, Langbein K, et al. Default mode network activity in schizophrenia studied at resting state using probabilistic ICA. *Schizophr Res.* 2012;138(2-3):143-149.
33. Erdeniz B, Serin E, İbadi Y, Taş C. Decreased functional connectivity in schizophrenia: The relationship between social functioning, social cognition and graph theoretical network measures. *Psychiatry Res Neuroimaging.* 2017;270:22-31.
34. Gawryluk JR, Palombo DJ, Curran J, Parker A, Carlsten C. Brief diesel exhaust exposure acutely impairs functional brain connectivity in humans: a randomized controlled crossover study. *Environ Health.* 2023;22(1):7.
35. Tripathi S. How does urbanization affect the human development index? A cross-country analysis. *Asia-Pacific Journal of Regional Science.* 2021;5:1053–1080.
36. Zhang Z, Zhao M, Zhang Y, Feng Y. How does urbanization affect public health? New evidence from 175 countries worldwide. *Front Public Health.* 2022;10:1096964.
37. Heinz A, Deserno L, Reininghaus U. Urbanicity, social adversity and psychosis. *World Psychiatry.* 2013;12(3):187-197.
38. Li X, Wei N, Song J, et al. The global burden of schizophrenia and the impact of urbanization during 1990-2019: An analysis of the global burden of disease study 2019. *Environ Res.* 2023;232:116305.
39. Fett AJ, Lemmers-Jansen ILJ, Krabbendam L. Psychosis and urbanicity: a review of the recent literature from epidemiology to neurourbanism. *Curr Opin Psychiatry.* 2019;32(3):232-241.
40. Robertson LJ. The impact of urbanization on mental health service provision: a Brazil, Russia, India, China, South Africa and Africa focus. *Curr Opin Psychiatry.* 2019;32(3):224-231.
41. Meng Q, Fang H, Liu X, Yuan B, Xu J. Consolidating the social health insurance schemes in China: towards an equitable and efficient health system. *Lancet.* 2015;386(10002):1484-1492.
42. Schneider M, Müller CP, Knies AK. Low income and schizophrenia risk: A narrative review. *Behav Brain Res.* 2022;435:114047.
43. Pratt GC, Vadali ML, Kvale DL, Ellickson KM. Traffic, air pollution, minority and socio-economic status: addressing inequities in exposure and risk. *Int J Environ Res Public Health.* 2015;12(5):5355-5372.
44. Zhang K, Batterman S. Air pollution and health risks due to vehicle traffic. *Sci Total Environ.* 2013;450-451:307-316.
45. End of 2017: the number of motor vehicles in China was 310 million and the number of drivers was 385 million [press release]. Beijing, China: the Xinhua News Agency, 01/15/2018 2018.
46. EPA. Air Quality Guide for Nitrogen Dioxide. In: Office of Air and Radiation, ed. Washington, D.C.: U.S. Environmental Protection Agency; 2011.

© 2024 Bai L et al. *JAMA Network Open.*
