# Supplementary material for: Ambient Air Pollution and Hospitalizations for Schizophrenia in China
Source: JAMA Netw Open. 2024 Oct 2;7(10):e2436915. doi: 10.1001/jamanetworkopen.2024.36915 (PMC11447564; doi:10.1001/jamanetworkopen.2024.36915)
Supplement: Supplement 2. — Data Sharing Statement [file jamanetwopen-e2436915-s002.pdf]

Data Sharing Statement

Bai. Ambient Air Pollution and Hospitalizations for Schizophrenia in China. *JAMA Netw Open*. Published October 02, 2024. doi:10.1001/jamanetworkopen.2024.36915

Data
Data available: No

Additional Information
Explanation for why data not available: Air pollution data can be available to others, but the health insurance data analyzed in this study are regulated by the governmental policies and cannot be made to the public due to privacy reasons.
